# Synthesis, In Silico Prediction and In Vitro Evaluation of Antimicrobial Activity, DFT Calculation and Theoretical Investigation of Novel Xanthines and Uracil Containing Imidazolone Derivatives

**DOI:** 10.3390/ijms222010979

**Published:** 2021-10-12

**Authors:** Samar El-Kalyoubi, Fatimah Agili, Wael A. Zordok, Ashraf S. A. El-Sayed

**Affiliations:** 1 Department of Pharmaceutical Organic Chemistry, Faculty of Pharmacy (Girls), Al-Azhar University, Nasr City, Cairo 11651, Egypt; 2 Chemistry Department, Faculty of Science (Female Section), Jazan University, Jazan 82621, Saudi Arabia; fatmah2000@gmail.com; 3 Department of Chemistry, Faculty of Science, Zagazig University, Zagazig 44519, Egypt; wazordok@zu.edu.eg; 4 Enzymology and Fungal Biotechnology, Botany and Microbiology Department, Faculty of Science, Zagazig University, Zagazig 44519, Egypt; ash.elsayed@gmail.com

**Keywords:** oxazolone, xanthine, 5,6-diaminouracil, imidazolone, schiff base, DFT calculation, antimicrobial, molecular docking

## Abstract

Novel xanthine and imidazolone derivatives were synthesized based on oxazolone derivatives **2a-c** as a key intermediate. The corresponding xanthine **3-5** and imidazolone derivatives **6-13** were obtained via reaction of oxazolone derivative **2a-c** with 5,6-diaminouracils **1a-e** under various conditions. Xanthine compounds **3-5** were obtained by cyclocondensation of 5,6-diaminouracils **1a-c** with different oxazolones in glacial acetic acid. Moreover, 5,6-diaminouracils **1a-e** were reacted with oxazolones **2a-c** in presence of drops of acetic acid under fused condition yielding the imidazolone derivatives **6-13**. Furthermore, Schiff base of compounds **14-16** were obtained by condensing 5,6-diaminouracils **1a,b,e** with 4-dimethylaminobenzaldehyde in acetic acid. The structural identity of the resulting compounds was resolved by IR, ^1^H-, ^13^C-NMR and Mass spectral analyses. The novel synthesized compounds were screened for their antifungal and antibacterial activities. Compounds **3**, **6**, **13** and **16** displayed the highest activity against *Escherichia coli* as revealed from the IC_50_ values (1.8–1.9 µg/mL). The compound **16** displayed a significant antifungal activity against *Candia albicans* (0.82 µg/mL), *Aspergillus flavus* (1.2 µg/mL) comparing to authentic antibiotics. From the TEM microgram, the compounds **3**, **12**, **13** and **16** exhibited a strong deformation to the cellular entities, by interfering with the cell membrane components, causing cytosol leakage, cellular shrinkage and irregularity to the cell shape. In addition, docking study for the most promising antimicrobial tested compounds depicted high binding affinity against acyl carrier protein domain from a fungal type I polyketide synthase (ACP), and Baumannii penicillin- binding protein (PBP). Moreover, compound **12** showed high drug- likeness, and excellent pharmacokinetics, which needs to be in focus for further antimicrobial drug development. The most promising antimicrobial compounds underwent theoretical investigation using DFT calculation.

## 1. Introduction

Increasing the incidence of microbial resistance to antibiotics due to the overuse and misuse of antimicrobial drugs is the major challenge for controlling the pathogenic bacteria. Recently, the World Health Organization reported that excessive use of antibiotics during the COVID-19 pandemic, as well as previous H_1_N_1_ influenza pandemics, increased the incidence of antimicrobial resistance [1,2,3]. Also, the infections by antibiotic -resistant bacteria has been exceeded that of tuberculosis, HIV infections and influenza combined [4,5]. As a result, patients of chronic diseases, cancer, organ transplantation, or other reasons are at the greatest risk of infection “difficult-to-treat” caused by antibiotic-resistant bacteria. Moreover, secondary bacterial infections are common in hospitalized patients, which can significantly worsen their prognosis [6,7,8], where it was revealed that one out of four patients had secondary bacterial infections [4,9].

In the last century, nitrogen-based heterocycles such as various imidazole-containing compounds have been implemented in clinical trials for their antimicrobial activity and treatment of variety of diseases. The rapid expansion of imidazole-based medicinal chemistry reveals the therapeutic potential of imidazole-derivatives in treatment of various incurable diseases. Comparison to other heterocyclic rings, the imidazole core scaffold has three carbon and two nitrogen atoms with electronic-rich characteristics with higher feasibility to bind with a variety of proteins and enzyme receptors, displaying strong broad spectrum biological activities such as anti-inflammatory and anticancer activities [1,2]. The antimicrobial activity of these imidazole containing compounds elaborates from their efficiency to cause DNA double-stranded break and inhibition of protein kinase. Moreover, the anti-inflammatory mechanisms of imidazole derivatives include inhibition of COX-2 enzyme, inhibition of neutrophils degranulation, and generation of reactive oxygen species [1,2]. The imidazole ring’s structural characteristics help to generate numerous drug–ligand interactions through van der Waals forces, hydrogen bonds and hydrophobic forces [10]. Furthermore, the imidazole core scaffold is naturally found in a variety of compounds, including histidine, histamine, alkaloids, biotin and nucleic acids and several FDA-approved medication classes [4]. Fused imidazole derivatives played a significant role in medicine due to their vital medical uses. Currently, the commercial imidazole derivatives with antimicrobial, anticancer and antiinflammatory activity are metronidazole, methotrexate and omeprazole, respectively [1]. Imidazolone derivatives are the most commonly exploited bioactive heterocycles. Functionalized imidazolones were recognized by their strong therapeutic effect, especially as a powerful CNS depressant, anticonvulsant, sedative and hypnotic, fungicidal, antibacterial, antihistamine, anti-inflammatory, MAO inhibitory, antihypertensive, antiparkinsonian and anthelmintic activities [4,5,6,7,8,9,11,12,13,14,15,16,17,18,19,20,21,22,23,24,25].

**Scheme 1:**
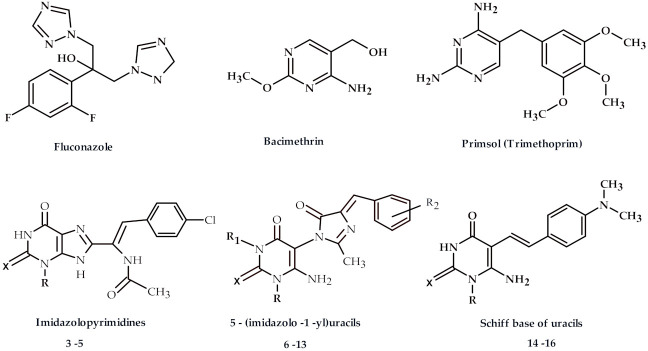
Chemical structures of Fluconazole, Bacmethrin, and Prismsol, as authentic drugs, and the current synthesized compounds “imidazolopyrimidine-2,4-diones, 5- (imidazole-1-yl) uracils and Schiff base of uracils.

Uracils and their derivatives are one of the most essential structural motifs for drug design [26]. The thymidine phosphorylase inhibitory action of substituted uracils at positions N1, N3, C5, and C6 is well recognized. They displayed a wide spectrum activity including antimicrobial activities [27,28,29,30], antiviral [31,32,33] and anticancer potency [34,35]. As well as, the acyclic and macrocyclic uracil derivatives with quaternized nitrogen exhibited a significant antibacterial and antifungal [36]. Interestingly, uracil derivatives were recognized as potent anticancer inhibitors by inhibiting histone deacetylase [37]. Based on this fact, new antimicrobial drugs will be needed to overcome these problems. Our strategy is directed towards synthesis of new compounds with potential activity against multidrug resistant Gram positive, and negative bacteria, in addition to human pathogenic fungi. The authentic drugs and the current synthesized compounds are showed in Scheme 1.

## 2. Results and Discussion

### 2.1. Chemistry

**Scheme 2:**
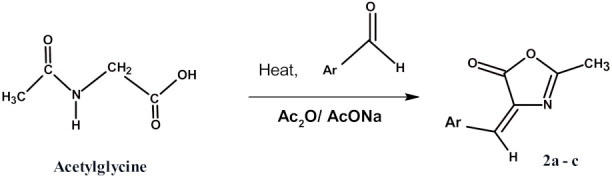
**2a**: Ar = C_6_H_5_, **2b**: 3-NO_2_.C_6_H_4_, **2c**: 4-Cl.C_6_H_4_.

Here, we reported the synthesis of novel purine derivatives and the assessment of their antibacterial and antifungal activities. 5,6-Diaminouracils **1a-e**, the key starting material for the obtained compounds, were synthesized by the reaction between ethyl cyanoacetate and different urea derivatives followed by nitrosation and reduction by conventional methods [25,38,39]. The Erlenmeyer azlactones (**2a-c**), also known as 4-arylideno-5(4*H*)-1,3-oxazolones, were synthesized according to literature [40,41,42,43] through an aldol-type condensation of a glycine-based azlactone (A) with different aldehydes (Scheme 2).

**Scheme 3:**
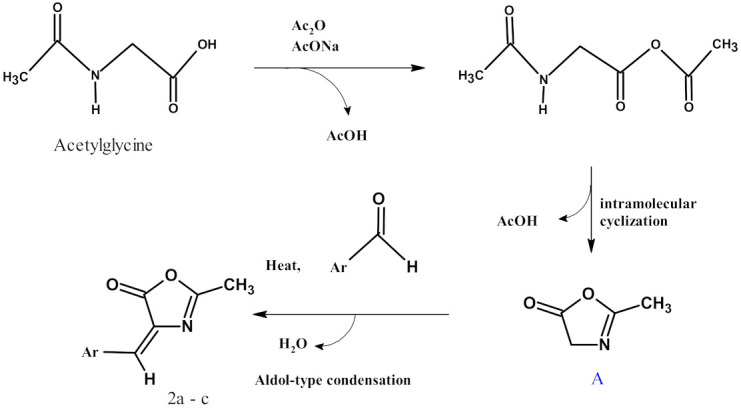
Mechanistic pathway for the synthesis of oxazolone derivatives **2a-c**.

The mechanistic pathway for the formation of azlactones **2a-c** was explained in (Scheme 3).

**Scheme 4:**
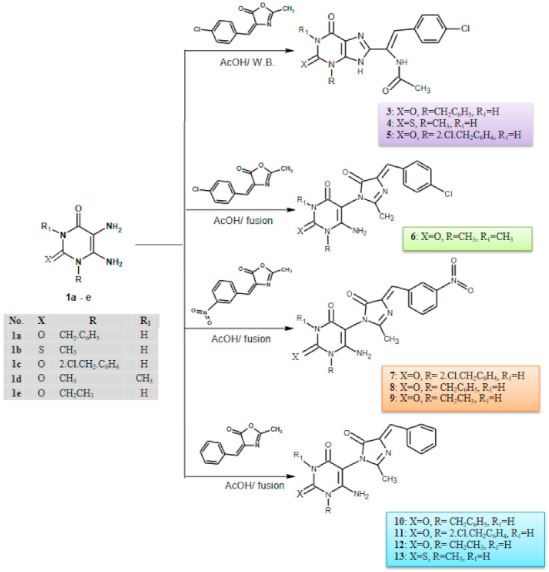
The reaction of uracils with different azlactones **3-13**.

Moreover, N-Substituted-8-purinyl-2-substituted vinylacetamide **3-5** were prepared by heating 5,6-diaminouracils **1a-c** in water bath with azlactone **2c** in acetic acid for 1h as illustrated in Scheme 4. The xanthine derivatives were crystallized from DMF/EtOH. Different analyses were applied to identify the resulting compounds. The most interesting observation in the ^1^H-NMR spectra of compounds **3-5** showed the disappearance of both signals of NH_2_ (5) and NH_2_ (6) around δ 5.0–6.0 ppm in compounds **1a-c** and the appearance of signals of NH (7) around δ 12.74–12.66 ppm, NH signals of acetamide group around δ 9.48–9.46 ppm, a singlet signal of CH benzylidene around δ 6.98–6.56 ppm and the upfield singlet signals of methyl group of acetamide around δ 1.91–1.90 ppm. While the IR spectra of the compounds **3-5** displayed a stretching band of NH within the range υ 3294–3278 cm^−1^ and the C=O groups (Amide I), red-shifted within the range υ 1705–1620 cm^−1^. ^13^C-NMR spectra indicates the formation of compounds **3-5** through the appearance of the CH benzylidene around δ 112 ppm and the upfield signals of methyl group of acetamide around δ 23 ppm.

Analogously, 5,6-diaminouracils **1a-e** were heated under fusion with different 1,3-oxazolones in acetic acid affording 6-amino-1-substituted-5- arylimidazolyluracil **6-13** in a high yield (Scheme 4). Compounds **6-13** are characterized by the disappearance of the NH_2_ (5) signals around δ 5.0–6.0 ppm, the presence of the NH_2_ (6) signals around δ 7.41–7.23 ppm, in addition to the appearance of singlet signals of CH benzylidene around δ 7.15–6.95 ppm and the upfield singlet signals of methyl group at δ 2.22–2.09 ppm. The IR spectra of compounds **6-13** showed the stretching band of NH within the range υ 3347–3172 cm^−1^ and the C=O groups (Amide I) within the range υ 1735–1627 cm^−1^, The strong asymmetric and symmetric NO_2_ stretching bands of the nitro group were noted at υ 1519–1512 and 1388–1381 cm^−1^. ^13^C-NMR spectra prove the formation of compounds **6-13** through the appearance of the CH benzylidene around δ 124.38–121.08 ppm and the upfield signals of methyl group at the range δ 16.41–15.04 ppm.

**Scheme 5:**
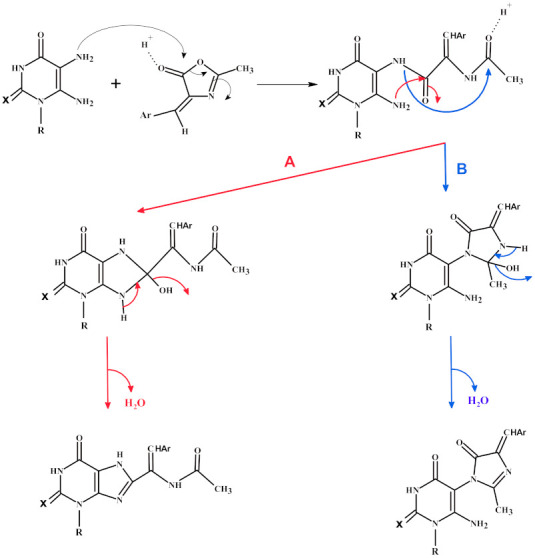
The suggested mechanism for the synthesis of xanthine and imidazolone derivatives.

Plausible mechanisms for these reactions are shown in Scheme 5 which takes place by two ways A or B. The acidic medium A first activates oxazolone carbonyl group via protonation which indicated by the blue arrows. Then, oxazolone ring is ruptured due to uracil amino group (5) attack. After that, pyrimidoimidazolone derivatives are obtained in the procedure of intramolecular cyclization B. Otherwise the amino group (6) attack the carbon of carbonyl group of oxazolone via protonation, then imidazolopyrimidine-2,4-diones were formed.

**Scheme 6:**
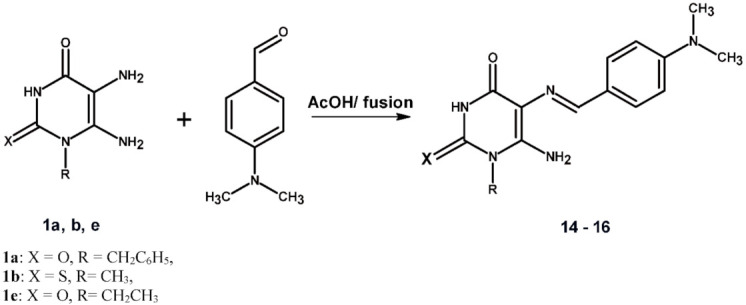
Formation of Schiff base.

Treatment of compounds **1a**,**b**,**e** with 4-dimethylaminobenzaldehyde in acetic acid was heated under fusion for 5-7 min to yield the target Schiff base derivatives **14-16** in high yields (Scheme 6). The compound **14** was synthesized by heating 5,6-diamino-1-methyl-2-thiouracil (**1b**) with 4-dimethylaminobenzaldehyde in dioxan at 80 °C with stirring for 2 h, as described by Nagamatsu [44]. Our improved method for synthesis of compound **14** has been conducted at 5–7 min, with 96% yield and affordable lower cost.

The structures of the synthesized Schiff base derivatives were confirmed on the basis of their IR, ^1^H-NMR, ^13^C-NMR, mass spectral data and elemental analysis.

### 2.2. Biological Activity

#### 2.2.1. Antimicrobial Activity

The antimicrobial efficacy of the synthesized uracil derivatives was evaluated by disc diffusion assay for the antibiotic resistant bacteria; *E. coli*, *Pseudomonas* sp., *Salmonella* sp., *Staphylococcus aureus* and *Bacillus* sp., in addition to pathogenic fungi (*A. flavus* and *C. albicans*). After desired incubation, the biological activity of the tested compounds was evaluated from the diameter of the inhibition zones. From the diameter of inhibition zones (Table 1), compounds **3** and **6** displayed the highest antifungal activity towards *C. albicans* (10 mm), *A. flavus* (2 mm), as well as antibacterial activity towards Gram positive bacteria *S. aureus* (1.0 mm) and Gram negative bacteria *E. coli* (4 mm). Practically, the compounds **3** and **6** exhibited the same physiological effect on the growth of bacteria and fungi revealing their chemical proximity. The compound **16** displayed the highest antifungal activity; *A. flavus* (10 mm) and *C. albicans* (12 mm), as well as highest activity against *E. coli* (7 mm), *Pseudomonas* sp. (1.9 mm) and *Bacillus* sp. (1.2 mm). The antibacterial and antifungal activities of the tested compounds were normalized to the authentic commercial antifungal (Fluconazole) and antibacterial (Bacimethrin, Primsol) drugs. As well as, the compounds # **11**, **12** and **13** exhibited a strong antifungal activity against *C. albicans*, *A. flavus* and *E. coli*. However, the compounds **4**, **5**, **7**, **8**, **10**, displayed a mild activity towards the tested microorganisms. Obviously, a fluctuation has been observed on the activity of the synthesized compounds towards the tested microorganisms, suggesting the structure-activity relationships of the synthesized compounds for each microorganism. DMSO at the 10% has been used as negative control, displaying no signs of antimicrobial activity towards the tested microorganisms.

**Figure 1:**
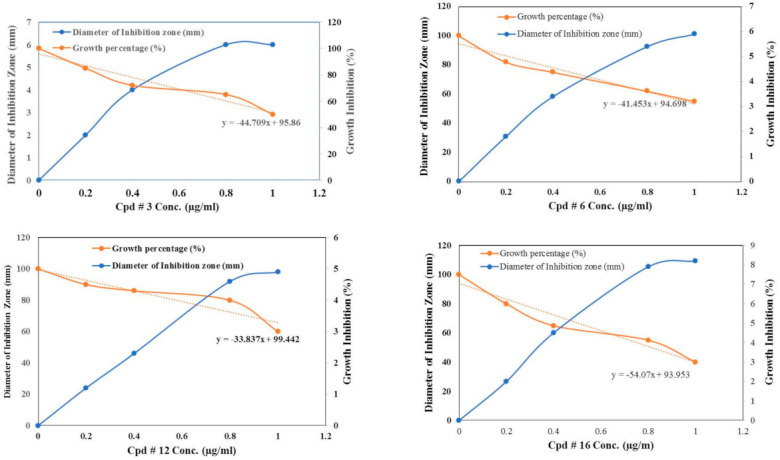
Kinetics of inhibition of the compounds **3**, **6**, **12** and **16** on *C. albicans*.

For further authentication of the antimicrobial activity of the tested compounds, different concentration of each compound was used, and after the desired incubation the kinetics of microbial growth of each microorganism was determined (Figure 1). The IC_50_ values for each compound against the tested microorganisms were summarized in Table 2. The compounds **3**, **6**, **12**, and **13** exhibited the highest activity against *E. coli* as revealed from the IC_50_ values that was 1.8–1.9 µg/mL. From the IC_50_ values, the compound **16** displayed a significant activity towards *C. albicans* (0.82 µg/mL), *A. flavus* (1.2 µg/mL), *E. coli* (1.9 µg/mL), *Salmonella* sp. (1.5 µg/mL), *Pseudomonas* sp. (2.1 µg/mL), *S. aureus* (2.6 µg/mL) and *Bacillus* sp. (2.1 µg/mL).

#### 2.2.2. TEM Analysis of Microorganisms in Response to the Tested Compounds

**Figure 2:**
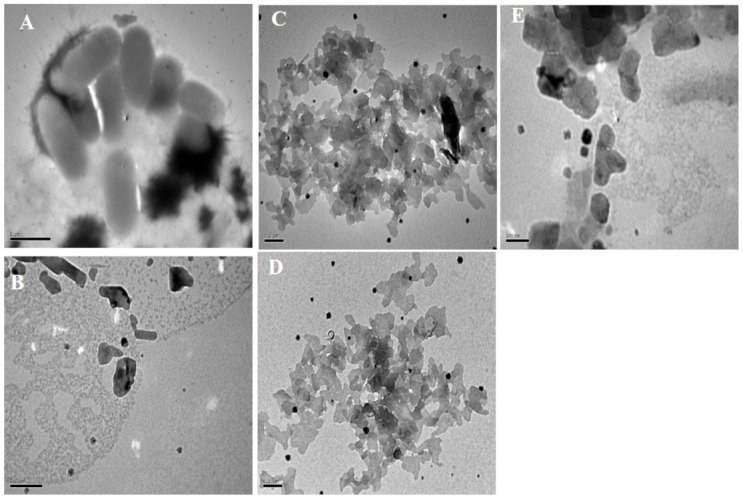
Effect of the tested compounds **3**, **12**, **13** and **16** on structural identity of *E. coli.* TEM Microgram of *E. coli* (Control, (**A**)) and presence of compounds **3** (**B**), compound **12** (**C**), compound **13** (**D**) and compound **16** (**E**).

**Figure 3:**
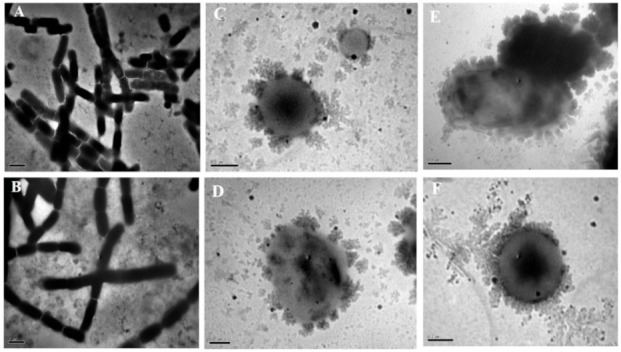
Effect of the tested compounds **3**, **12**, **13**, **14** and **16** on structural identity of *Pseudomonas* sp. TEM Microgram of *Pseudomonas* sp. (Control, (**A**)) and presence of compounds **3** (**B**), compound **12** (**C**), compound **13** (**D**) and compound **14** (**E**) and compound **16** (**F**).

**Figure 4:**
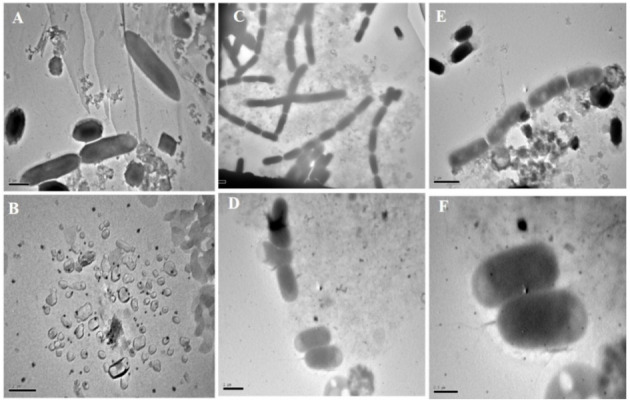
Effect of the tested compounds **3**, **12**, **13**, **14** and **16** on structural identity of *Klebsiella* sp. TEM Microgram of *Klebsiella* sp. (Control, (**A**)) and presence of compounds **3** (**B**), compound 12 (**C**), compound **13** (**D**) and compound **14** (**E**) and compound **16** (**F**).

The cytomorphological deformations of the microorganisms in response to the tested compounds were evaluated by TEM. The effects of the most potent uracil derivative-compounds on the cellular structure of Gram positive and Gram-negative bacteria were assessed. The influence of the most potent compounds on the cellular structures of *E. coli* was assessed (Figure 2). An obvious cellular deformation on the cell wall and cell membrane was observed upon treatment with the compounds **3**, **12**, **13**, **14** and **16**. With treatment of *E. coli* by the compounds at their MIC values, a complete distortion and ruptures to the bacilli type, comparing to regular bacillus shape of control. The cellular structure of *Pseudomonas* sp., upon treatment with the compounds **3**, **12**, **13**, **14,** and **16** was evaluated by TEM. From the results of TEM analysis (Figure 3), a complete morphological deformation to bacterial cells from short rod cells into rounded cells, was observed. A complete rupture on the cell walls and cell membranes was observed, with obvious leakage to the cellular organelles, caused by the partial ruptures on the cell walls. The rationality of destructive effect of the compound **16** may be due to the suppression of pivotal biological secondary metabolites especially the proper cellular morphogenesis as revealed from the binding affinity with ACP domain of polyketide synthase type I. The cellular deformation of the *Klebsiella* sp., in response to the compounds **3**, **12**, **13**, **14** and **16** has been observed (Figure 4), a complete distortion to the cellular identity of *Klebsiella* in response to treatment with these compounds. From the TEM micrograph, a dramatic deformation has been noticed to the bacterial cell walls and various organelles in response to the tested compounds. A remarkable alternations and deformation to the cells, irregularity on shape, loss of symmetry, equivocal distribution of the cytoplasmic organelles, especially the cell wall and plasma membrane that becoming a highly denser, have been observed. The powerful antibacterial activity of the compounds **12** and **16** elaborates from their higher binding affinity with the ACP domains of PKS type I [45] and DNA gyrase [46], blocking the synthesis of essential secondary metabolites and DNA replication. Increase on the plasma membrane permeability causes a strong cracking to the bacterial cell leading to a visual release of the cytoplasmic materials. With the treatment, almost all cellular organelles were strongly deforming, in contrary to the control cells which are usually short bacilli, with a multilayered cell surface consisting of an outer membrane, a peptidoglycan layer in the periplasmic space, the cytoplasmic membrane was relatively uniform [47].

#### 2.2.3. Cytotoxicity Analysis

##### Cytotoxicity Assay

The cytotoxicity of the experimental compounds was evaluated towards VERO-E6 cells using the (MTT) assay. As revealed from the half maximal cytotoxic concentration (CC_50_), the tested compounds **3**, S-Aza **1**, **5**, **6**, **8**, **11**, **12**, **13**, **14**, **9** displayed relatively no signs of toxicity in which the CC50 values were ranged from 160 to 676 µg/mL as shown from the Supplementary Material (Figure S4). Negative control of 10% DMSO has been used. The DMSO (10%) did not display any signs of toxicity to the VERO-E6 cells.

### 2.3. Molecular Docking Study

To take one step further, and to determine the mode of action of the tested compounds as potential anti-microbial agents, molecular-docking study was employed to determine the binding modes against three important proteins implicated in fungal and bacterial targeting mechanism including APC, DNAg, and PBP. These targets were selected based on structure activity relationship with the corresponding co-crystalized ligands with our tested compounds. The cocrystal ligand for the three proteins (PNS for APC protein, CBN for DNAg protein, and PNM for PBB protein) were redocked to assure the validity of the docking parameters and methods to represent the position and orientation of the ligand detected in the crystal structure. The difference of RMSD value between cocrystal ligands to the original cocrystal ligand was <2 Å which approved the accuracy of the docking protocols and parameters.

**Figure 5:**
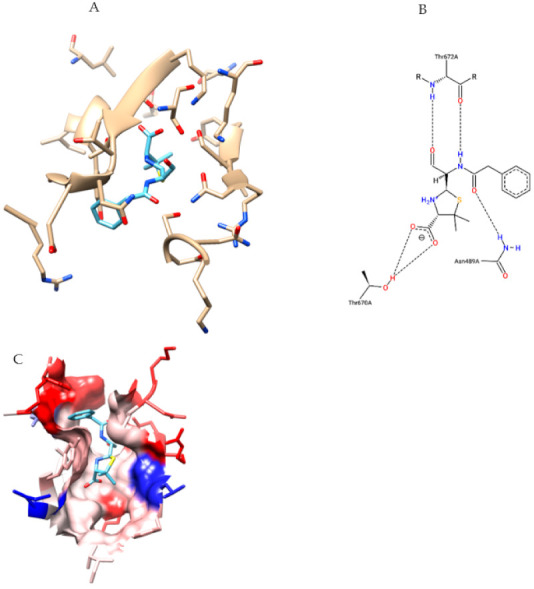
Reference ligand interaction with PBP protein, (**A**) 3D interaction, (**B**) hydrogen bond formation, (**C**) Hydrophobic interaction (represented by blue color).

**Figure 6:**
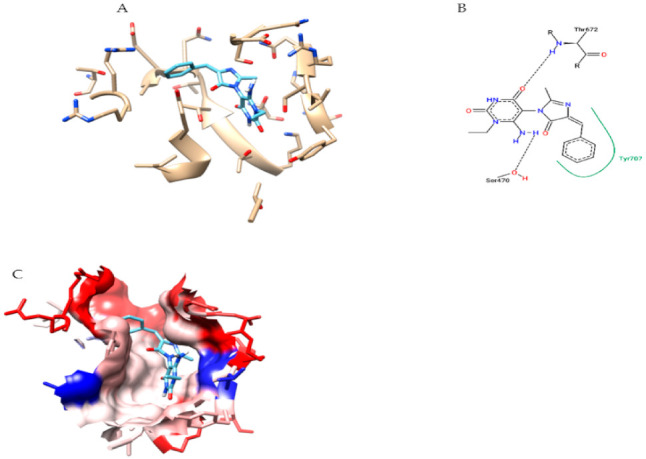
Compound **12** interaction with PBP protein, (**A**) 3D interaction, (**B**) hydrogen bond formation, (**C**) Hydrophobic interaction (represented by blue color).

To elucidate the mode of action of the most promising antimicrobial compounds “**3**, **12**, **13**, and **16**” docking studies were conducted. Compounds **3**, **12**, **13** showed an affinity to bind with **APC** protein active site, that was evidenced by low binding energy compared to reference ligand 4’-Phosphopantetheine (**PNS**) (−5.1, −5.1, −5.2 Kcal/mol, respectively), while compound **16** has the same binding free energy value of the reference ligand (−4.5 Kcal/mol) (Table 3). All tested compounds formed one hydrogen bond with key amino acid residue Glu65 (Table 3). Docking results against DNAg protein, showed less binding affinity of all tested compounds against this protein, which was evident by high free of energy for all compounds compared to reference ligand Clorobiocin (**CBN**) (Table 3). In addition, docking score against PBP protein, showed a favorable binding affinity to this protein, as revealed from the low values of energy of free binding compared to reference ligand penicillin G (**PNG**) (Table 3). Compounds **3**, and **12** showed the lowest values binging free energybinging free energy (−8.7, and −8.2 Kcal/mol), compared to **PNG** (−6.7 Kcal/mol), moreover compound **3** formed one hydrogen bond interaction with amino acid (ASN674: 2.006 Å), while compound **12** formed three hydrogen bonds with the key amino acid residues in the PBP pocket (SER470: 2.118; SER434: 3.269; THR672: 2.797) (Table 3), and (Figure 5 and Figure 6).

### 2.4. Pharmacokinetics and ADME Activity

**Figure 7:**
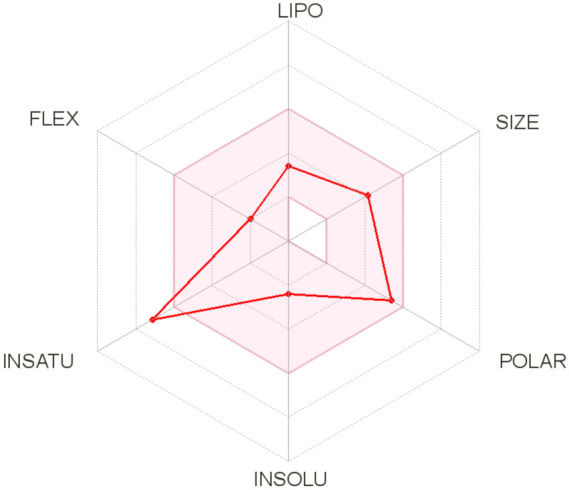
The Bioavailability Radar shows that compound **12**, represented by central red lines fits in the pink area which is the optimal range for each property.

**Figure 8:**
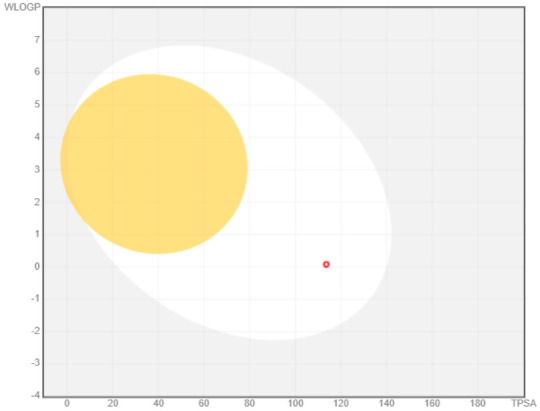
Pharmacokinetics bioled egg model. Yellow colour represents blood brain barrier (BBB), while colour represents gastrointestinal absorption for the selected drugs. Red circle represents compound **12**.

To predict the physicochemical and drug-likeness properties, bioinformatics study for the compound **12** was carried out (Table 4). Bioavailability Radar has been generated to check the suitable physicochemical properties for oral bioavailability, based on 6 characters including (lipophilicity: XLOGP3 between −0.7 and +5.0, size: *M*_W_ between 150 and 500 g/mol, polarity: TPSA between 20 and 130 Å, solubility: log S not higher than 6, saturation: fraction of carbons in the sp^3^ hybridization not less than 0.25, and flexibility: no more than 9 rotatable bonds) (Table 4, Figure 7). Compound **12** exhibited good bioavailability, and good water solubility, while it showed good pharmacokinetics with high gastrointestinal absorption (GI), and it is does not permit blood brain barrier (BBB) as it depicted in the boiled Egg model (Figure 8). In addition, compound **12** has good drug likeness properties according to Lipinski rule of five for drug discovery without any violation.

### 2.5. Computational Details

#### 2.5.1. Structural Parameters and Models

N-(1-(3-benzyl-2,6-dioxo-2,3,6,7-tetrahydro-1H-purin-8-yl)-2-(4-chlorophenyl)vinyl) acetamide, compound **3**. The activity of hetero cyclic compounds is mainly determined by its structure, this compound has many characteristic structural features. The bond length N22-C21 is 1.395 Å, C21-N0 is 1.389 Å, there is a single bond characters C and N atoms [48], the bond lengths of N17-C10 and N11-C10 are 1.269 and 1.339 Å, respectively, whereas the N17-C10 bond has double bond characters, while the N11-C10 has single bond characters [48]. Detailed analysis of corresponding bond lengths in various hetero cyclic compounds were given elsewhere [49]. All distances and angles between the atoms of the ligand I was given in (Table 5). The C21-O24 bond length is 1.208 Å, while the C19-O23 bond length is 1.210 Å [50], C13- N12 is 1.384 Å [49] and the C13=O14 bond length of carbonyl group is 1.209 Å [50].

**Figure 9:**
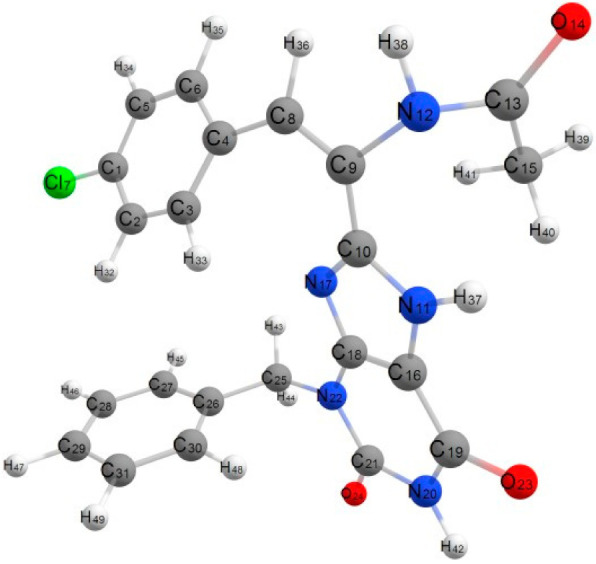
Optimized geometrical structure of compound (**3**), N-(1-(3-benzyl-2,6-dioxo-2,3,6,7 -tetrahydro-1*H*-purin-8-yl)-2-(4-chlorophenyl)vinyl)acetamide by using DFT calculations.

The molecule is a highly sterically-hindered, there are three aromatic rings. The molecule is non-planar, there are two plans one occupied by the two aromatic rings and other plane occupied by the purine ring and also, the two plans are perpendicular respect to each other. The purine ring is lying out of plane of the plane occupied by the two aromatic rings, the dihedral angles C26C25N22C18 is −84.71° ≈ 90° and the acetamide group is lying in perpendicular plane respect to purine ring system, the C10C9N12C13 is 82.58° ≈ 90°, where the values are neither zero nor 180°. The C10N17C18N22, 178.05° ≈ 180°, also the dihedral angles: N22C25C26C27, −175.85° ≈ 180° and N22C25C26C20, −4.19° ≈ 0.00°, where the values are nearly zero or 180°. Figure 9, the terminal benzyl group is lying in perpendicular plane respect to purine ring system, with a dihedral bond angle C8C9N10N17 is 102.31° ≈ 90°, Figure 9 shows the optimized geometrical structure of the compound (3), N-(1-(3-benzyl-2,6-dioxo-2,3,6,7-tetrahydro-1H-purin-8-yl)-2- (4-chlorophenyl)vinyl)acetamide, the dihedral angles O23C19N20C21 is 178.85° ≈ 180° and O24C21N20C19 is −179.53° ≈ 180°, which confirms that the O23 and O24 are lying in the same plane of the nitrogen atom N20 of purine ring. The oxygen atom O14 of acetamide group is lying in trans position respect to the C9 which bonded with purine ring with dihedral angle 168.98° ≈ 180°, while the others two oxygen atoms O23 and O24 are lying in the same plane away from the plane occupied by O14 of acetamide group, this confirms that one oxygen atoms of these three atoms can be chelated as donor with any acceptor according to the value of accumulated charge density lying on it. The value of bond angle C4C8C9 is 131.89°, C26C25N22 is 113.98°, C8C9C10 is 126.94° and C9N12C13 is 124.18° reflects on sp2 hybridization on the C8, C25, C9 and N12.

There is building up of charge density on the hetero atoms, nitrogen and oxygen atoms, the highest charged atoms are O14 of carbonyl group in acetamide with −0.488, while the others two oxygen atoms O23 and O24 have lower charge density values, −0.446 and −0.432, so the active site in this compound is O14 as set in docking study in biological investigation, the compound **3** favors bonding with any receptor through the oxygen atom O14 of carbonyl group. The accumulated charge density on nitrogen atoms N11, N17, N20, N22 and N12 involved in this compound are −0.107, −0.334, −0.432, −0.198 and −0.284, respectively. The value of energy of this compound is −172,059.722 kcal/mol, heat of formation of this compound is −10,607.049 kcal/mol and slightly weak dipole 3.078D. Detailed analysis of corresponding bond lengths in various hetero cyclic compounds were given elsewhere [51,52]. All distances and angles between the atoms of the ligand are given in (Table 5).

##### 6-Amino-5-(4-benzylidene-2-methyl-5-oxo-4,5-dihydro-1H-imidazol-1-yl)-1-ethylpyrimidine-2,4(1H,3H)-dione, compound **12**

The bond length C5-N4 is 1.381Å, C5-N6 is 1.388 Å and the N13-C17 bond length is 1.368 Å, so there is a single bond characters C and N atoms [48], the bond lengths of N16-C17 is 1.352 Å, whereas this bond has a double bond characters [48]. Detailed analysis of corresponding bond lengths in various hetero cyclic compounds were given elsewhere [49]. All bond distances and angles between the atoms of the compound **12**, 6-amino-5-(4-benzylidene-2-methyl-5-oxo-4,5-dihydro-1*H*-imidazol-1-yl)-1-ethylpyrimidine-2,4(1*H*,3*H*)-dione are given in (Table S1). The C3-O7 and C5-O8 bond lengths of the pyrimidine ring are 1.211 Å and 1.208 Å [50], while the C14-O18 of the imidazole ring is 1.209 Å.

**Figure 10:**
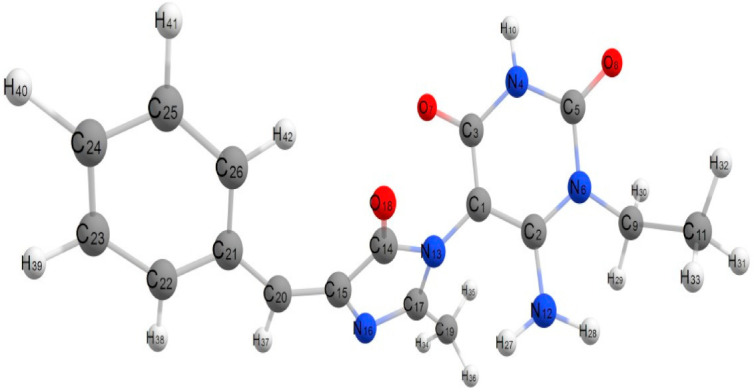
Optimized geometrical structure of compound (**12**), 6-amino-5-(4-benzylidene-2-methyl-5-oxo-4,5- dihydro-1*H*-imidazol-1-yl)-1-ethylpyrimidine-2,4(1*H*,3*H*)-dione by using DFT calculations.

The pyrimidine ring is lying out of plane of the plane occupied by the others two aromatic rings, the dihedral angles C17N13C1C3 and C17N13C1C2 are 109.33° ≈ 90° and −70.47° ≈ 90°, respectively, where the values are neither zero nor 180˚. These values confirm that the pyrimidine ring is lying in perpendicular plane respect to the central aromatic imidazole ring of the compound. Also, the value of dihedral angle of C5N6C9C11 is 90.54° ≈ 90°, this value confirms that the terminal ethyl group is lying in perpendicular plane respect to the pyrimidine ring. The dihedral angle N16C15C20C21 is 171.89° ≈ 180°, also the dihedral angle, C15C20C21C22 is 176.18° ≈ 18°, these values confirm that the benzylidene ring is lying in the same plane of the imidazole ring, also the values of the dihedral angles C26C21C20C15 and C21C20C15C14 are 7.63° ≈ 0.00° and 4.55° ≈ 0.00° as shown in Figure 10. The main observation from the dihedral angles of the optimized geometrical structure of the compound **12** that the two oxygen atoms O7 and O8 are lying in the same plane, while the oxygen atom O18 is lying out of this plane, one of these atoms can be participate in the chelation as donor atom with any acceptor atom. The calculated mullkine charge density over these oxygen atoms helps us to detect which of these oxygen atoms can be behaved as active site in bonding with acceptor atom in docking calculations.

There is building up of charge density on the all hetero atoms nitrogen and oxygen atoms, the highest negative charged atoms are O7 and O8, −0.442 and −0.432, while the negative charges on nitrogen atoms of the pyrimidine ring, N4 and N6 are −0.328 and −0.208, also the negative charges on the nitrogen atoms of imidazole ring, N13 and N16 are −0.135 and −0.385. These values of charges confirmed that the oxygen atoms can be participate in bonding with any acceptor more than nitrogen atoms involved in the compound **12**, so the oxygen atom, O7 can be considered as active site in this compound and can bond through it with any acceptor as shown in the docking calculations. The charge density on other atoms less negative than these atoms as listed in Table S1. The energy of the compound **12**, 6-amino-5-(4-benzylidene-2-methyl-5-oxo- 4,5-dihydro-1*H*- imidazol-1-yl)-1-ethylpyrimidine-2,4(1*H*,3*H*)-dione is−141,856.224 kcal/mol, heat of formation is −8661.756 kcal/mol and slightly high dipole 8.326D. From the value of energy we found that this compound has higher energy value greater than the compound **3**, which has lower energy and lower dipole moment.

##### 6-Amino-5-(4-benzylidene-2-methyl-5-oxo-4,5-dihydro-1H-imidazol-1-yl)-1-methyl-2-thioxo-2,3-dihydropyrimidin-4(1H)-one, compound **13**

**Figure 11:**
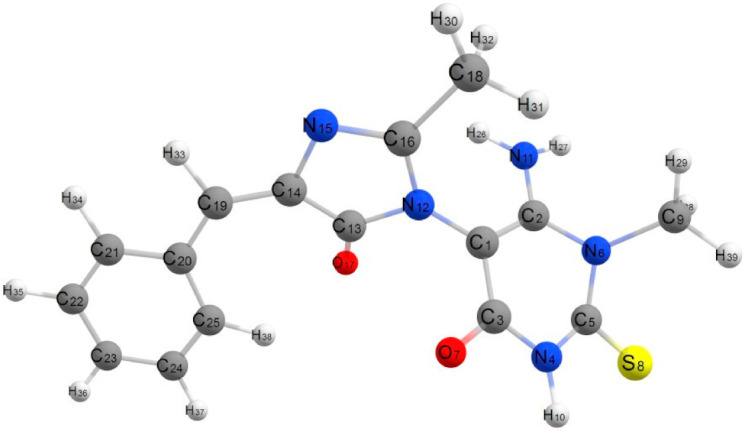
Optimized geometrical structure of compound **13**, 6-amino-5-(4-benzylidene-2-methyl-5-oxo- 4,5-dihydro-1*H*-imidazol-1-yl)-1-methyl-2-thioxo-2,3-dihydropyrimidin-4(1*H*)-one by using DFT calculations.

This compound is similar to the compound (12) with replacement of the C=O of the pyrimidine ring with C=S, the bond length C3-N4 is and C5-N4 are 1.369 and 1.383 Å, there is a single bond characters C and N atoms in the pyrimidine ring [48], also the bond lengths of N12-C16, C13-N12 and C14-N15 of the imidazole ring are 1.352, 1.348 and 1.359 Å, these values reflect that these bonds have single bond characters, while the bond distance of C16-N15 of the imidazol ring is 1.339 Å, whereas this bond has double bond characters [48] as shown in the (Figure 11). All calculated bond distances, bond angles and dihedral angles between the atoms of the compound **13** are given in (Table S2). The C5-S8 bond length is 1.692 Å [53] and the C3-O7 bond length is 1.209 Å [50], while the C13-O17 bond length is 1.208 Å [50].

There is no significant difference between the structure of this compound and compound (**12**), the benzylidene ring and dihydropyrimidine ring are lying in the same plane, the dihedral angles C13C14C19C20 and C14C19C20C25 are 6.42° ≈ 0.00° and 11.35° ≈ 0.00°, these values confirm that the benzylidene ring and dihydropyrimidine ring are lying in the same plane, while the dihedral angle C3C1N12C13 is −49.18, so the imidazol ring is lying out of the plane occupied by others two rings, as shown in the (Figure 11). The dihedral angles C2N6C5S8 is −153.03° and O7C3C1C2 is 169.59˚, which confirms that the O7 and S8 are lying in the same plane of the nitrogen atom N4 of dihydropyrimidin ring, while the dihedral angle O17C13N12C1 of the imidazol ring is 79.9° ≈ 90.0°, so the oxygen atom O17 is lying away from the other plane occupied by O7 and S8. In this compound there are many hetero atoms can behave as active donating site, the active donating site can be detected from the value of accumulated charges spreading over hetero atoms.

There is building up of negative charge density on the nitrogen atoms, oxygen atoms and sulfur atom in the compound **13**, 6-amino-5-(4-benzylidene-2-methyl-5- oxo-4,5-dihydro-1*H*-imidazol-1-yl)-1-methyl-2-thioxo-2,3-dihydropyrimidin-4(1*H*)-one, oxygen atom, O7 and sulfur atom, S8 are the highest charged atoms, −0.413 and −0.616, while nitrogen atoms have lower charge densities less than oxygen atoms, the charge accumulated on N4 (−0.389) and N6 are (−0.261) and (−0.163), while the charge on the N12 and N15 are −0.134 and −0.347. The charge density on other atoms less negative than these atoms as listed in Table S3, so that the compound **13** have a power full chelating sites oxygen atom, O7 and sulfur atom S8 more than others compounds, from the optimized structure the bonding of compound **13** favors bonding through the oxygen atom O7 not through sulfur atom S8, the dihedral angles C2N6C5S8 and C9N6C5S8 are 153.03° and −43.49°, these values reflected that sulfur atom is not completely planer, while the dihedral angles N12C1C3O7 and O7C3C1C2 are −13.93° ≈ 00.0° and 169.59° ≈ 180.0°, these values reflected that oxygen atom O7 is nearly planer, so oxygen atom, O7 is considered as active site in bonding with acceptor atom. The energy of the compound **13** is −132,805.565 kcal/mol, heat of formation is −8086.449 kcal/mol and highly dipole 8.586 D more than others previous compounds.

##### 6-Amino-5-((4-(dimethylamino)benzylidene)amino)-1-ethylpyrimidine-2,4(1H,3H)-dione, compound (**16**)

This compound is more energetic than all studied compound, the energy of it is −123,990.259 kcal/mol, so this compound is less stable than others. The heat of formation of this compound is −8026.826 kcal/mol, the value of dipole moment is 6.051 D, this value is slightly high, this value is greater than dipole moment of the compound **3** and less than compounds **12** and **13**, this result attributed to the presence of C25=N24 bond as bridge between the two aromatic rings, which facilitates the rotation of any aromatic ring around it. All geometrical parameters of the compound **16**, 6-amino-5- ((4-(dimethylamino) benzylidene)amino)-1-ethylpyrimidine -2,4(1*H*,3*H*)- dione, bond lengths, bond angles and dihedral angles are given in (Table S3). The bond length C16-N15 is 1.379 Å is longer slightly than bond length of C14-N15 is 1.376 Å, from the values of bond lengths, C13-N17 and N17-C16, there is a single bond characters C and N atoms [49], the bond length of C25-N24 is 1.347 Å, whereas this bond has a double bond characters [49]. Detailed analysis of corresponding bond lengths in various hetero cyclic compounds were given elsewhere [50]. The C14-O18 and C16-O19 bond lengths of the pyrimidine ring are 1.211 Å and 1.208 Å [50].

**Figure 12:**
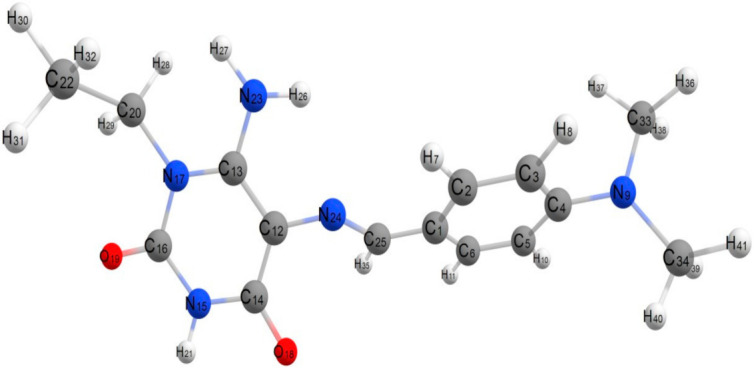
Optimized geometrical structure of compound (**16**), 6-amino-5-((4-(dimethylamino) benzylidene)amino)- 1-ethylpyrimidine-2,4(1*H*,3*H*)-dione by using DFT calculations.

As seen in (Figure 12) of the optimized geometrical structure of the compound **16**, The compound is non planar and there are two planes each of them ia perpendicular respect to each other, the dihedral angles C25N24C12C14 and C25N24C12C13 are 50.26˚ and −131.88°, where the values are neither zero nor 180°, these values reflected that the plane occupied by pyrimidine ring is perpendicular with the plane occupied by benzylidene ring, the dihedral angle C13N17C20C22 is −83.59° ≈ 90°, this value confirms that the terminal ethyl group is lying in plane perpendicular with the plane occupied by pyrimidine ring. Also, the value of dihedral angle of C5N6C9C11 is 90.54° ≈ 90°, this value confirms that the terminal ethyl group is lying in perpendicular plane respect to the pyrimidine ring. The dihedral angles C20N17C16O19 and N24C12C14O18 are 1.65° ≈ 0.00° and 0.10° ≈ 0.00° as shown in (Figure 12). The main observation from the dihedral angles of the optimized geometrical structure of the compound **16** that the two oxygen atoms O18 and O19 are lying in the same plane, one of them can be considered as an active site in this compound. The calculated mullkine charge density over these two oxygen atoms can be used to detect which of them can be used in bonding with acceptor atom in docking calculations.

There is building up of charge density on the all hetero atoms nitrogen and oxygen atoms, the highest negative charged atoms are O18 and O19, −0.448 and −0.459, while the negative charges on nitrogen atoms of the pyrimidine ring, N15 and N17 are −0.429 and −0.203, also the negative charge on the terminal nitrogen atom of amino group, N9 is −0.437. These values of charges confirmed that the oxygen atom, O18 can behave as an active site in the compound **16** and can bond through it with any acceptor as shown in the docking calculations. The charge density on other atoms less negative than these atoms as listed in (Table S4).

#### 2.5.2. Charge Distribution Analysis

The charge distribution analysis on the optimized geometry configuration of the all studied compounds was made on the basis of natural population analysis (NPA). The charge distribution on the hetero atoms, nitrogen, oxygen and sulfur of the different studied compounds indicates the presence of a net negative pole attributed to the presence of polar atoms beside the high degree of planarity of these molecules, as a result the molecules are a slightly high dipole in case of compound **3**, 8.586D, while compound **2** has slightly weak dipole, 3.078D less than others compounds. There is building up of charge density on the donating oxygen, nitrogen and sulfur atoms involved in the studied compounds. All calculated parameters, dipole moment, mulliken charges and total energies are given in (Table 6). From the factors affecting on the degree of stability of these compounds is the value of the total energy and distribution charges over all atoms in compounds. From the values of energies, the compound **3**, N-(1-(3-benzyl-2,6-dioxo- 2,3,6,7-tetrahydro-1*H*-purin-8-yl)-2-(4-chlorophenyl)vinyl)acetamide is considered as more stable than others, with more negative energy value less than second compound **12** by 30,203.498 kcal/mol, while the third compound in stability is **13**, the difference in energy between it and the first compound **3** is 39,254.157 kcal/mol. The energy difference between the less stable compound **16** and the most stable compound **3** is 48,069.463 kcal/mol.

#### 2.5.3. Molecular Orbitals and Frontier

Molecular orbitals play also an important role in the electric properties, as well as in UV-Vis [54]. An electronic system with smaller values of HOMO-LUMO gap should be more reactive than one having a greater energy gap [55]. The energy gap, ΔE of the studied compounds varied between 0.061 for Compound **13** which more reactive and 0.082 eV for compound **3** which less reactive, so electron movement between these orbitals could be easily occur by decreasing the value of energy gap, so that there is a peak around 250 nm in the UV-Vis spectra for all studied compounds as shown in UV-Vis spectra for all studied compounds. The energy gap is closely associated with the reactivity and stability of the executed compounds and shows the nature of the compound with low kinetic stability and slightly high chemical reactivity. On the other hand, the adjacent orbitals are often closely spaced on the frontier region.

**Figure 13:**
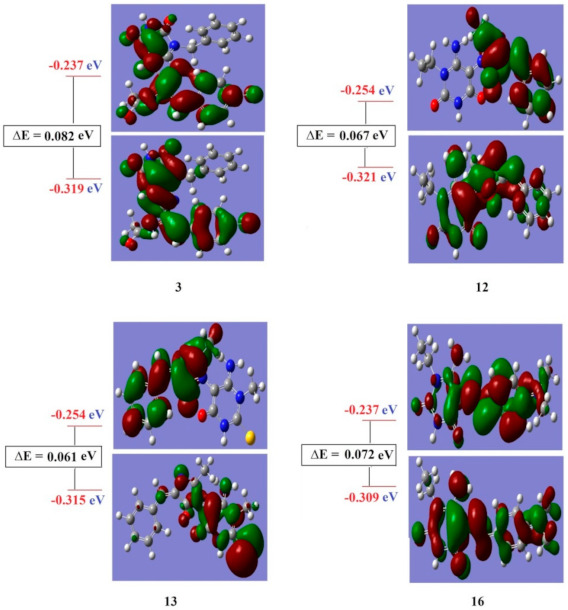
Molecular orbital surfaces and energy levels of all studied compounds, **3**, **12**, **13** and **16** by using DFT calculations.

The nodal properties of molecular orbitals of studied complexes in Figure 13 are illustrative and suggest orbital delocalization, strong orbital overlap, and low number of nodal planes. The energy difference between HOMO and LUMO (energy gap, ∆E) for the all studied compounds varied according to the composition of each compound or the type of substitutions as shown in (Table 7), and (Figure 13), shows the isodensity surface plots of HOMO and LUMO for the studied compounds. Hard molecules have high the HOMO-LUMO gap, and soft molecules have smaller HOMO-LUMO gap [56]. The values of η and ∆E (HOMO-LUMO) are given in (Table 7). It is obvious that the compound **3** is considered as most hard with η equals to 0.041, and compound **13** is considered as less hard molecule with η equals to 0.031, also the electronic transition within the compounds is easy as indicated from the ∆E. There are some quantum chemical parameters depending upon the energy values of HOMO and LUMO were calculated as global softness (S), electro negativity (χ), absolute softness (σ), chemical potential (Pi), global electrophilicity (ω) and additional electronic charge (∆N_max_) of the all studied compounds. From these values, the compound **3** is absolute more soft according to the (σ = 24.390 eV), while the compound **13** is treated as less soft compound (σ = 32.258 eV).

As seen in Figure 13, the electron density of HOMO, Ψ 113 of the compound **3** is delocalized over all atoms with highly percent on the two oxygen atoms of the C=O groups of the 2,6-dioxopurine ring with percent 55.5%, with spreading of the remaining percentage on the remaining components, with 20.2% on the acetamide group, 15.4 % on the chlorophenyl ring, 7.9% on the vinyl group and finally 1% on the benzyl group. The LUMO, Ψ 114, the electron density delocalized over all atoms of compound **3**, the most percent (53.7%) is localized on the 2,6-dioxopurine ring with spreading of remaining percentage over the remaining components except benzyl group with 0.0%, as given in (Table 8). Also the HOMO, Ψ 89 of the compound **12**, the electron density delocalized over all atoms, the most percent (61.2%) is localized on the 2-methyl-5-oxoimidazol ring and 25.6% on the 2,4-dioxo-6-aminopyrimidine ring with small percent 13.2% on the benzylidene ring, without any percent on the terminal ethyl group. The LUMO, Ψ 90 of the compound **12**, the electron density delocalized over all atoms, the most percent (51.9%) is localized on the 2,4-dioxo-6-aminopyrimidine ring and 48.1% on the benzylidene ring only. 

The HOMO of the compound **13**, Ψ 89, the electron density localized over 2-thio-4-oxo-6-aminopyrimidine ring with percent (56.3%) and 41.9% on the 2-methyl-5-oxoimidazol ring, with small percent on benzylidene ring (1.8%) and without any percent on the terminal methyl group. The LUMO, Ψ 90, the electron density delocalized over all atoms, the most percent (52.7%) is localized on the 2-methyl-5-oxoimidazol ring and 31.4% on the benzylidene ring with small percent on the terminal methyl group (15.9%), while 0.0% on the 2-thio-4-oxo-6-aminopyrimidine.

In compound **16**, the HOMO, Ψ 80, the electron density delocalized over all atoms of this compound with percent (41.3%) on the 2,4-dioxo-6-aminopyrimidine group, 33.6% on the dimethylaminobenzylidene ring and 25.1% on the Amino group with 0.0% on the terminal ethyl group. The LUMO, Ψ 81, the electron density delocalized over all atoms in this compound with 58.7% localized on the dimethylaminobenzylidene ring, 21.8% on the amino group and 19.5% on the 2,4-dioxo-6-aminopyrimidine ring without any percent on the ethyl group 0.0%. The variation in the distribution of electron densities on the components of HOMO and LUMO for each compound play important role in the variation of electronic transitions occurred in compounds and effect on the degree of biological effect of them.

#### 2.5.4. Excited State

The TD-DFT at the B3LYP level by using G98W program proved to give accurate description of the UV-vis. spectra [57,58]. Time-dependent density functional response theory (TD-DFT) has been recently reformulated [59] to compute discrete transition energies and oscillator strengths and has been applied to a number of different atoms and molecule. Bauernschmitt and Ahlrichs [60] included hybrid functional proposed in the calculation of the excitation energies. These hybrid methods typically constitute a considerable improvement over conventional Hatree-Fock (HF) based methods. In this work, the optimized geometry was calculated and was used in all subsequent calculations; the wave functions of SCF MOs were explicitly analyzed. The calculated wave functions of the different MOs reflect and suggest the fraction of the different fragments of the compounds contributing to the total wave functions of different states. The results indicate that there is an extent of electron delocalization in the different molecular orbitals.

The electronic transition could be described as a mixed n → π* and π → π* transitions. The energies of HOMO and LUMO states for the all studied compounds are listed in Table 8. The HOMO can perform as an electron donor and the LUMO as the electron acceptor in reaction profile.

Table 8 indicates that there are six excited states are involved in case of compound **3**, N-(1-(3-benzyl-2,6-dioxo-2,3,6,7-tetrahydro-1*H*-purin-8-yl)-2-(4-chlorophenyl)vinyl) acetamide, the first exited state results from a combination eight transitions, H-11 → L(4.5%), H-11 → L + 6(2.9%), H → L(4.9%), H-7 → L(3.2%), H-5 → L(34.5%), H-3 → L(3.7%), H-1 → L(14.7%), H → L(4.4%), this excited state assigned to π-π* at 250.24 nm and 4.9546 eV, this theoretical value agree with experimental peak at 265 nm. The second excited state result from the interaction between electronic configurations which represent π → π* transition. This is transition results from H-11 → L (7.8%), H-10 → L (2.5%), H-5 → L (2.8%), H-1 → L (78.4%), H → L (4,6%), H → L + 1(3.6%), the energy of transition is 4.2308 eV, calculated wave length is 258.98nm and can be observed experimentally at 275 nm. The third excited state is assigned to n → π* transition, represents a transition appears at 280.861 nm and 4.4144 eV, H-11 → L + 7 (4.9%), H-10 → L + 6 (14.1%), H-10 → L + 7 (41.8%), H-7 → L + 7 (6.7%), H-6 → L + 6 (9.6%), H-6 → L + 7 (26.7%). The fourth excited state at 302.43 nm and 4.0996 eV, results as H-9 → L (5.2%), H-9 → L + 5 (61.6%), H-9 → L + 6 (21.3%), H-9 → L + 7 (2.7%), H-8 → L + 5(6.3%), H-8 → L + 6 (2.8%) and assigned to π-π*. The fifth excited state at 356.3 nm and 3.4797 eV, results as H-11 → L + 2 (5.3%), H-10 → L (6.3%), H-10 → L + 1 (10.4%), H-6 → L (22.9%), H-6 → L + 1 (38.3%), H-6 → L + 7 (8.8%), H-6 → L + 10 (7.8%) and assigned to n-π*. The sixth excited state is assigned to π → π* transition, represented at 377.47nm and 3.2847 eV, H-1 → L (4.5%), H → L (93.4%). All calculated transitions agree with experimental data obtained from the UV-Visible spectra as given in Table 8. Also, in compound **13**, 6-amino-5- (4-benzylidene-2-methyl-5-oxo-4,5-dihydro-1H-imidazol-1-yl)-1-ethylpyrimidine-2,4(1H,3H)-dione, the first excited state assigned to an π → π* transition at 258.65 nm and 4.7935 eV, this transition represented by H-8 → L + 1 (2.4%), H-1 → L + 1 (54.4%), H → L + 1 (43%). The second excited state is assigned to π → π* transitions, represents a transitions H-6 → L (5.8%), H-4 → L (9.8%), H-3 → L (8.6%), H-2 → L (2.7%), H-1 → L (72.8%) which observed around 280.61 nm and 4.4183 eV. The third excited state is assigned to n → π* transition, represents a transition appears at 285.12 nm and 4.3485 eV, H-9 → L + 3 (20.5%), H-9 → L + 4 (12.4%), H-8 → L + 3 (7.6%), H-8 → L + 4 (4%), H-7 → L + 3 (27.3%), H-3 → L + 4 (16.6%), H-5 → L + 3 (7%), H-5 → L + 4 (4.2%). The fourth excited state at 308.64 nm and 4.0172 eV, results as H-10 → L (5%), H-8 → L (35.2%), H-8 → L + 3 (3.3%), H-8 → L + 4 (7.3%), H-3 → L (15.4%), H-3 → L + 3 (8.5%), H-3 → L + 4 (8.2%), H-3 → L + 5 (7%) and assigned to n-π* transitions. The fifth excited state is assigned to n → π* transition, represents a transition appears at 322.23 nm and 3.8482 eV, H-6 → L + 1 (6.1%), H-5 → L + 1 (35.8%), H-5 → L + 3 (13.5%), H-5 → L + 6 (13.6%), H-4 → L + 1 (18.3%), H-4 → L + 3 (6.3%), H-4 → L + 6 (6%). The sixth excited state at 332.23 nm and 3.7319eV, results as one transition only H → L (100%) and assigned to π-π* transitions. The seventh excited state at 342.43 nm and 3.6207 eV, results as H-8 → L (14.5%), H-8 → L + 5 (8.9%), H-6 → L (3.3%), H-5 → L (11.7%), H-5 → L + 5 (3%), H-4 → L (5.7%), H-3 → L (36.7%), H-3 → L + 5 (3.5%) and assigned to n-π * transitions.

As given in Table 8, the experimental data of the UV-Visible spectra of compound **3**, **12** are compared nicely with calculated excited states.

In compound **13**, there are five peaks obtained in spectra, while theoretically there are nine transitions can be observed as given in (Table S4). The first peak obtained at 267 nm, this peak is composed of two theoretical transition states overlapped together at 260.77 nm and 263.14 nm, these transitions are assigned to π-π *. The second peak obtained at 290 nm, we found that this peak is consist of two overlapped excited states at 284.13 nm and 284.13 nm, the first one is assigned to π-π * and the second is assigned to n-π * transitions. Also, the third peak observed at 322 nm is composed of three overlapped transition states at 288.71 nm, 326.03 and 328.13 nm, the first and third transition states assigned to π-π * transitions, while the second transition states assigned to n-π* transition. The fourth peak observed experimentally at 339 nm and this peak is agree theoretically with these electronic transitions, H-10 → L (2.8%), H-9 → L (12.7%), H-9 → L + 4 (8.1%), H-6 → L (9.7%), H-6 → L + 5 (8.5%), H-5 → L (4.1%), H-5 → L + 5 (10.7%), H-4 → L (82.6%), H-2 → L + 2 (17.3%), which obtained at 339.43 nm and 3.7876 eV, this transition is assigned to n-π*. The last peak observed experimentally at 357nm, this peak can be represented by these transitions, H-2 → L + 1 (3.2%), H-3 → L (7.8%), this excited state is assigned to n-π * transition, the theoretical wave length corresponding with excited state is 359.12 nm with energy equals to 3.4525 eV.

In (Table S5), the number of excited states involved in compound **16** are five transition states, there are four of them are assigned to π-π *, which can be observed at 249.89, 313.44, 335.42 and 365.71nm, while fourth excited state is assigned to n-π *, this transition can be observed at 278.83 nm.

## 3. Material and Methods

### 3.1. Chemistry

#### 3.1.1. Materials and Instruments

All melting points were determined by an Electrothermal Mel.-Temp. II apparatus and were uncorrected. Element analyses were performed at Regional Center for Mycology and Biotechnology at Al-Azhar University. The infrared (IR) spectra were recorded using potassium bromide disc technique on Nikolet IR 200 FT IR (Perkin-Elmer, Akron, OH, USA). Mass spectra were recorded on DI-50 unit of Shimadzu GC/MS-QP 5050A at the Regional Center for Mycology and Biotechnology at Al-Azhar University. The proton nuclear magnetic resonance (^1^H-NMR) spectra were recorded on Bruker 400 MHz Spectrometer and ^13^C-NMR spectra were run at 125 MHz in dimethylsulfoxide (DMSO-*d*6) and TMS as an internal standard, Applied Nucleic Acid Research Center, Zagazig University, Egypt. All new compounds gave corresponding elemental analyses (C, H, N, typically ±0.3%). All reactions were monitored by TLC using precoated plastic sheets silica gel (Merck 60 F_254_) and spots were visualized by irradiation with UV light (254 nm). The used solvent system was chloroform: methanol (9:1) and ethyl acetate: toluene (1:1).

#### 3.1.2. Synthetic Procedures

5,6-diaminouracils **1a-e.** These compounds were prepared according to a reported method [25,38,39]. 4-arylideno-5(4*H*)-1,3-oxazolones (**2a-c**) These compounds were prepared according to a reported method [40,41,42,43].

#### 3.1.3. N-Substituted-8-purinyl-2-(arylvinyl)acetamides **3**-**5**

General method: A mixture of 5,6-diaminouracils (0.75 mmol) and the appropriate azalactone derivatives (0.75 mmol) in acetic acid (3 mL) was heated in a water bath for 1 h. The formed precipitate was filtered, washed with ethanol and recrystallized from DMF/ethanol (2:1).

#### 3.1.4. N-(1-(3-Benzyl-2,6-dioxo-2,3,6,7-tetrahydro-1H-purin-8-yl)-2-(4-chlorophenyl)vinyl) acetamide (3)

Yield: 80%; m.p. = 220 °C; IR (KBr) ν_max_ (cm^−1^): 3291, 3194 (NH), 3032 (CH arom.), 2924 (CH aliph.), 1705, 1651 (C=O), 1581 (C=N), 1512 (C=C), 833 (*p*-substituted phenyl); ^1^H NMR (DMSO-d_6_) δ 12.71 (s, 1H, NH), 10.69 (s, 1H, NH), 9.48 (s, 1H, NH), 8.13–8.10 (d, *J* = 8.4 Hz, 1H, arom.), 7.62–7.58 (dd, *J* = 8.4 Hz, 2H arom.), 7.47–7.45 (d, *J* = 8.4 Hz, 2H, arom.), 7.38–7.31 (m, 3H, arom.), 7.29–7.19 (m, 1H, arom.), 6.56 (s, 1H), 5.09 (s, 2H), 1.90 (s, 3H, CH_3_); ^13^C NMR (DMSO-d_6_): δ 171.88, 167.50, 161.56, 154.65, 151.54, 136.48, 135.19, 133.33, 132.36, 132.31, 129.88, 129.83, 128.58, 127.66, 127.39, 88.14, 45.46, 23.54; MS: m/z (%) = M + 2, 437 (55), M+, 435 (34), 430 (58), 426 (77), 425 (74), 400 (55), 347 (94), 320 (100), 313 (65), 279 (70), 253 (66), 171 (54), 162 (97), 54 (74), 53 (98); Anal. calcd. for C_22_H_18_ClN_5_O_3_ (435.87): C, 60.62; H, 4.16; N, 16.07; Found: C, 60.76; H, 4.28; N, 16.32.

#### 3.1.5. N-(2-(4-Chlorophenyl)-1-(3-methyl-6-oxo-2-thioxo-2,3,6,7-tetrahydro-1H-purin-8-yl) vinyl) acetamide (**4**)

Yield: 74%; m.p.=314 °C; IR (KBr) ν_max_ (cm^−1^): 3309, 3201 (NH), 3039 (CH arom.), 2924 (CH aliph.), 1666, 1620 (C=O), 1550 (C=N), 1489 (C=C), 818 (*p*-substituted phenyl); ^1^H NMR (DMSO-d_6_): δ 12.49 (s, 1H, NH), 12.04 (s, 1H, NH), 9.67 (s, 1H, NH), 7.99–7.97 (d, *J* = 8.0 Hz, 2H), 7.62–7.60 (d, *J* = 8.0 Hz, 2H), 6.79 (s, 1H), 3.73 (s, 3H), 1.93 (s, 3H) ppm.; ^1^H NMR (DMSO-d_6_): δ 175.76, 172.87, 158.90, 158.32, 154.78, 151.16, 145.10, 134.12, 129.76, 124.18, 107.12, 92.64, 37.12, 23.37 ppm.; MS: m/z (%) = M + 2, 377 (36), M+, 375 (35). 340 (80), 332 (38), 300 (36), 214 (39), 211 (37), 201 (32), 198 (30), 197 (57), 180 (100), 178 (43), 168 (73), 167 (48), 166 (41), 164 (59), 155 (58), 153 (55), 145 (52), 140 (40), 135 (54), 132 (45), 128 (64), 126 (44), 124 (86), 91 (71); Anal. calcd. for C_16_H_14_ClN_5_O_2_S (375.83): C, 51.13.62; H, 3.75; N, 18.63; Found: C, 51.40; H, 3.89; N, 18.91.

#### 3.1.6. N-(1-(3-(2-Chlorobenzyl)-2,6-dioxo-2,3,6,7-tetrahydro-1H-purin-8-yl)-2-(4-chlorophenyl) vinyl)acetamide (**5**)

Yield: 64%; m.p.=260–263 °C; IR (KBr) ν_max_ (cm^−1^): 3294, 3178 (NH), 3070 (CH arom.), 2978 (CH aliph.), 1705, 1620, (C=O), 1566 (C=N), 1489 (C=C), 825 (*p*-substituted phenyl), 756 (*o*-substituted phenyl); ^1^H NMR (DMSO-d_6_): δ 12.66 (s, 1H, NH),11.04 (s, 1H, NH), 9.48 (s, 1H, NH), 7.63–7.31 (d, *J* = 8.4 Hz, 1H, arom.), 7.52- 7.50 (d, *J* = 8.8 Hz, 1H arom.), 7.41-7.39 (d, *J* = 8.8 Hz, 1H, arom.), 7.37-7.33 (m, 1H, arom.), 7.30-7.29 (m, 1H, arom.), 7.19–6.99 (m, 3H, arom.), 6.68 (s,1H.), 5.12 (s, 2H), 1.91 (s, 3H, CH_3_) ppm.; ^1^H NMR (DMSO-d_6_): δ 168.45, 159.70, 154.46, 150.21, 148.49, 144.84, 141.15, 138.11, 136.19, 133.57, 132.11, 130.80, 129.94, 129.28, 128.04, 125.85, 125.55, 124.31, 121.86, 80.50, 43.80, 34.10 ppm; MS: m/z (%) = M + 4, 474 (32), M + 2, 472 (35), M+, 470 (52), 463 (34), 447 (41), 220 (74), 211 (68), 162 (54), 54 (100), 52 (55); Anal. calcd. for C_22_H_17_Cl_2_N_5_O_3_ (470.31): C, 56.18; H, 3.64; N, 14.89.

#### 3.1.7. 1-Substituted-6-amino-5-(4-arylidene-2-methyl)imidazolylpyrimidinediones **6**-**13**

A mixture of 5,6-diaminouracils (0.75 mmol)**,** the appropriate oxazole derivatives (0.75 mmol) and drops of acetic acid was heated under fusion for 5–8 min. An adequate amount of ethanol was added to the residue. The precipitate was filtered, washed with ethanol and crystallized from DMF/ethanol (2:1).

#### 3.1.8. 6-Amino-5-(4-(4-chlorobenzylidene)-2-methyl-5-oxo-4,5-dihydro-1H-imidazol-1-yl)-1,3-dimethylpyrimidine-2,4-(1H,3H)-dione (**6**)

Yield: 74%; m.p. > 330 °C; IR (KBr) ν_max_ (cm^−1^): 3317, 3170 (NH_2_), 3065 (CH arom), 2951 (CH aliph), 1712, 1674 (C=O), 1621 (C=C), 1566 (C=N); ^1^H NMR (DMSO-d_6_): 9.67 (s, 1H, benzylidenic), 7.93-7.90 (d, *J* = 8.0 Hz, 2H, arom.), 7.44-7.41 (d, *J* = 8.0 Hz, 2H, arom.), 7.37 (s, 2H, NH_2_), 3.39 (s, 3H, CH_3_), 3.16 (s, 3H, CH_3_), 2.06 (s, 3H, CH_3_); ^13^C NMR (DMSO-d_6_): δ 160.61, 159.70, 155.14, 151.05, 150.00, 147.70, 144.97, 134.51, 131.92, 130.22, 129.32, 105.32, 31.01, 30.03, 18.44 ppm. MS: m/z (%) = M + 2, 375 (17), M+, 373 (29), 357 (43), 305 (18), 290 (100), 277 (35), 238 (51), 186 (74), 129 (14), 79 (23), 49 (42); Anal. calcd. for C_17_H_16_ClN_5_O_3_ (373.09): C, 54.65; H, 4.31; N, 18.74;. Found: C, 54.89; H, 4.50; N, 18.95.

#### 3.1.9. 6-Amino-1-(2-chlorobenzyl)-5-(2-methyl-4-(3-nitrobenzylidene)-5-oxo-4,5-dihydro-1H-imidazol-1-yl)pyrimidine-2,4(1H,3H)-dione (**7**)

Yield: 65 %; m.p.=206 °C; IR (KBr) ν_max_ (cm^−1^): 3340, 3201 (NH_2_), 3062 (CH arom.), 2931 (CH aliph.), 1705, 1635 (C=O), 1581 (C=N), 1514 (C=C), 1512 (NO_2_ asymstr), 1381 (NO_2_ symstr), 771 (*m*-substituted phenyl), 748 (*o*-substituted phenyl); ^1^H NMR (DMSO-d_6_) δ 11.14 (s, 1H, NH, exchangeable), 9.21 (s, 1H, arom.), 8.62–8.60 (d, *J* = 8.0 Hz, 1H, arom.), 8.25–8.22 (d, *J* = 8.0 Hz, 1H, arom.), 7.78–7.74 (m, 1H, arom.), 7.53- 7.51 (m, 1H, arom.), 7.41–7. 7.35 (m, 2H, arom. & 2H NH_2_, exchandeable), 7.15 (s, 1H), 6.99–6.97 (m, 1H, arom.), 5.13 (s, 2H), 2.22 (s, 3H, CH_3_); ^13^C NMR (DMSO-d_6_): δ 170.66, 168.54, 159.70, 150.21, 148.49, 141.29, 139.30, 138.11, 136.36, 136.19, 133.57, 132.06, 130.00, 129.35, 127.92, 125.81, 125.72, 125.55, 124.34, 121.81, 43.84, 22.83 ppm.; MS: m/z (%) = M + 2, 482 (25), M+, 480 (35), 475 (30), 455 (47), 436 (46), 335 (54), 300 (40), 298 (53), 227 (56), 225 (46), 220 (100), 153 (75), 128 (79), 60 (59), 53 (98); Anal. calcd. for C_22_H_17_ClN_6_O_5_ (480.87): C, 54.95; H, 3.56; N, 17.48;. Found: C, 55.12; H, 3.71; N, 17.69.

#### 3.1.10. 6-Amino-1-benzyl-5-(2-methyl-4-(3-nitrobenzylidene)-5-oxo-4,5-dihydro-1H-imidazol-1-yl)pyrimidine-2,4(1H,3H)-dione (**8**)

Yield: 68%; m.p. = 178 °C; IR (KBr) ν_max_ (cm^−1^): 3363, 3201 (NH_2_), 3070 (CH arom.), 2924 (CH aliph.), 1705, 1635, (C=O), 1573 (C=N), 1527 (C=C), 1512 (NO_2_ asymstr), 1381 (NO_2_ symstr), 771 (*m*-substituted phenyl); ^1^H NMR (400 MHz, DMSO-d6) δ 11.08 (s, 1H, NH), 9.20 (s, 1H, arom.), 8.61–8.59 (d, *J* = 7.8 Hz, 1H, arom.), 8.24–8.22 (d, *J* = 8.4 Hz, 1H, arom.), 7.76–7.68 (m, 3H, arom.), 7.38–7.23 (m, 5H, arom. & NH_2_), 7.14 (s, 1H), 5.15 (s, 2H), 2.16 (s, 3H, CH_3_); ^13^C NMR (DMSO-d_6_): δ 167.59, 159.30, 153.96, 150.28, 148.14, 140.94, 137.69, 136.11, 130.24, 128.61, 128.54, 127.30, 126.27, 126.17, 125.57, 123.86, 121.21, 82.97, 44.37, 21 ppm.; MS: m/z (%) = M+, 446 (31), 442 (40), 439 (36), 431 (67), 409 (80), 480 (69), 375 (54), 366 (57), 352 (57), 348 (66), 161 (100), 77 (73); Anal. calcd. for C_22_H_18_N_6_O_5_ (446.42): C, 59.19; H, 4.06; N, 18.83; Found: C,58.97; H, 4.23; N, 18.97.

#### 3.1.11. 6-Amino-1-ethyl-5-(2-methyl-4-(3-nitrobenzylidene)-5-oxo-4,5-dihydro-1H-imidazol-1-yl) pyrimidine-2,4(1H,3H)-dione (**9**)

Yield: 89%; m.p. = 298–300 °C; IR (KBr) ν_max_ (cm^−1^): 3327, 3232 (NH_2_), 3085 (CH arom.), 2993 (CH aliph.), 1735, 1651 (C=O), 1558 (C=N), 1473 (C=C), 1519 (NO_2_ asymstr), 1388 (NO_2_ symstr), 771 (*m*-substituted phenyl); ^1^H NMR (DMSO-d_6_) δ 10.95 (s,1H, NH exchangeable), 9.20 (s, 1H arom.), 8.60-8.58 (d, *J* = 8.0 Hz, 1H, arom.), 8.24–8.22 (dd, *J* = 8.2 Hz, 1H, arom.), 7.78–7.74 (t, *J* = 8.0 Hz, 1H, arom.), 7.28 (s, 2H, NH_2_ exchangeable), 7.13 (s, 1H), 3.92–3.87 (q, *J* = 6.9 Hz, 2H), 2.14 (s, 3H, CH_3_), 1.16–1.13 (t, *J* = 6.9 Hz, 3H). ^13^C NMR (DMSO-d_6_): δ 170.14, 167.67, 159.46, 153.52, 150.05, 148.18, 141.02, 137.68, 136.04, 130.20, 125.54, 123.80, 120.89, 83.25, 37.09, 15.22, 13.00 ppm.; MS: m/z (%) = M+, 384 (24), 374 (26), 358 (48), 313 (64), 300 (78), 290 (100), 285 (48), 283 (92), 238 (73), 200 (57), 152 (52), 139 (53), 56 (66); Anal. calcd. for C_17_H_16_N_6_O_5_ (384.35): C, 53.12; H, 4.20; N, 21.87; Found: C, 53.41; H, 4.37; N, 21.75.

#### 3.1.12. 6-Amino-1-benzyl-5-(4-benzylidene-2-methyl-5-oxo-4,5-dihydro-1H-imidazol-1-yl)pyrimidine-2,4(1H,3H)-dione (**10**)

Yield: 76%; m.p. = > 320 °C; IR (KBr) ν_max_ (cm^−1^): 3325, 3178 (NH_2_), 3050 (CH arom.), 2930 (CH aliph.), 1697, 1666, (C=O), 1559 (C=N), 1496 (C=C); ^1^H NMR (DMSO-d_6_) δ 11.06 (s,1H, NH), 8.24-8.22 (d, *J* = 7.3 Hz, 2H, arom.), 7.48–7.45 (t, *J* = 7.3 Hz, 2H, arom.), 7.42–7.36 (m, 3H, arom.), 7.31–7.24 (m, 5H, arom. & 2H NH_2_), 6.95 (s, 1H), 5.14 (s, 2H), 2.12 (s, 3H, CH_3_); ^13^C NMR (DMSO-d_6_): δ 170.38, 165.56, 159.28, 153.87, 150.27, 139.16, 136.20, 134.31, 131.73, 129.77, 128.72, 128.57, 127.31, 126.16, 124.06, 83.26, 44.63, 14.88 ppm.; MS: m/z (%) = M+, 401 (35), 386 (20), 291 (40), 249 (100), 232 (80), 231 (74), 229 (57), 170 (48), 401 (35); Anal. calcd. for C_22_H_19_N_5_O_3_ (401.43): C, 65.83; H, 4.77; N, 17.45; Found: C,66.04; H, 4.93; N, 17.66.

#### 3.1.13. 6-Amino-5-(4-benzylidene-2-methyl-5-oxo-4,5-dihydro-1H-imidazol-1-yl)-1-(2-chlorobenzyl)pyrimidine-2,4(1H,3H)-dione (11)

Yield: 79%; m.p = 318 °C; IR (KBr) ν_max_ (cm^−1^): 3340, 3201 (NH_2_), 3062 (CH arom.), 2931 (CH aliph.), 1705, 1635, (C=O), 1581 (C=N), 1512 (C=C), 749 (*o*-substituted phenyl); ^1^H NMR (DMSO-d_6_): δ 11.11 (s, 1H, NH), 8.25–8.23 (d, *J* = 7.3 Hz, 2H, arom.), 7.53–7.51 (dd, *J* = 7.5 Hz, 1H, arom.), 7.49- 7.43 (t, *J* = 7.3 Hz, 2H, arom.), 7.41–7.36 (m, *4*H, arom.), 7.34 (s, 2H), 6.96 (s, 1H), 5.13 (s, 2H), 2.17 (s, 3H, CH_3_); ^13^C NMR (DMSO-d_6_): δ 170.39, 165.65, 159.35, 154.00, 149.98, 139.21, 134.32, 133.59, 131.73, 131.67, 129.87, 129.59, 128.76, 128.72, 127.54, 125.25, 124.24, 83.41, 43.73, 15.17 ppm. MS: m/z (%) = M + 2, 437 (11), M+, 435 (27), 390 (16), 388 (41), 183 (59), 180 (72), 160 (29), 158 (77), 88 (55), 69 (100); Anal. calcd. for C_22_H_18_ClN_5_O_3_ (435.87): C, 60.62; H, 4.16; N, 16.07; Found: C,60.85; H, 4.38; N, 16.31.

#### 3.1.14. 6-Amino-5-(4-benzylidene-2-methyl-5-oxo-4,5-dihydro-1H-imidazol-1-yl)-1-ethylpyrimidine-2,4(1H,3H)-dione (**12**)

Yield: 86%; m.p. = 287–280 °C; IR (KBr) ν_max_ (cm^−1^): 3347, 3181 (NH_2_), 3028 (CH arom.), 2921 (CH aliph.), 1720, 1631, (C=O), 1573 (C=N), 1513 (C=C); ^1^H NMR (DMSO-d6): δ 10.88 (s, 1H, NH), 8.25–8.23 (d, *J* = 7.3 Hz, 2H, arom.), 7.46–7.44 (t, *J* = 7.3 Hz, 2H, arom.), 7.42–7.38 (m, *J* = 7.3 Hz, 1H, arom.), 7.24 (s, 2H), 6.95 (s,1H), 3.90-3.87 (q, *J* = 7.0 Hz, 2H), 2.09 (s, 3H), 1.16–1.12 (t, *J* = 7.0 Hz, 3H); ^13^C NMR (DMSO-d_6_): δ 170.47, 165.69, 159.21, 153.53, 149.89, 139.24, 134.34, 131.72, 129.74, 128.71, 123.96, 83.26, 37.06, 15.18, 13.00 ppm. MS: m/z (%) = M+, 339 (33), 335 (28), 307 (50), 278 (76), 261 (56), 259 (78), 250 (70), 181 (100), 151 (61), 149 (58), 148 (69) 144 (55), 140 (80), 137 (65), 339 (34), 127 (53), 52 (51); Anal. calcd. for C_17_H_17_N_5_O_3_ (339.36): C, 60.17; H, 5.05; N, 20.64; Found: C,60.43; H, 5.21; N, 20.87.

#### 3.1.15. 6-Amino-5-(4-benzylidene-2-methyl-5-oxo-4,5-dihydro-1H-imidazol-1-yl)-1-methyl-2-thioxo-2,3-dihydropyrimidin-4(1H)-one (**13**)

Yield: 82%; m.p = 308–310 °C; IR (KBr) ν_max_ (cm^−1^): 3317, 3178 (NH_2_), 3072 (CH arom.), 2931 (CH aliph.), 1697, 1627, (C=O), 1559 (C=N), 1496 (C=C); ^1^H NMR (DMSO-d_6_) δ 12.36 (s, 1H), 8.25–8.23 (d, *J* = 7.2 Hz, 2H, arom.), 7.49–7.39 (m, 3H, arom. & NH_2_), 6.98 (s, 1H), 3.77 (s,3H, CH_3_), 2.13 (s, 3H, CH_3_); ^13^C NMR (DMSO-d_6_): δ 176.08, 170.11, 164.93, 156.80, 153.99, 139.05, 134.24, 131.78, 129.87, 128.74, 124.38, 87.85, 36.29, 15.08 ppm.; MS: m/z (%) = M+, 341 (27), 337 (43), 332 (44), 294 (40), 290 (50), 264 (66), 241 (51), 216 (41), 165 (51), 149 (48), 108 (51), 97 (93), 88 (100), 56 (80); Anal. calcd. for C_16_H_15_N_5_O_2_S (341.39): C, 56.29; H, 4.43; N, 20.51; Found: C,56.41; H, 4.52; N, 20.68.

#### 3.1.16. 1-Substituted-6-amino-5-((4-(dimethylamino)benzylidene)aminopyrimidindiones **14**-**16**

A mixture of 5,6-diaminouracils (0.75 mmol)**,** 4-(dimethylamino)benzaldehyde (0.75 mmol) and drops of acetic acid was heated under fusion for 5–7 min. An adequate amount of ethanol was added to the residue. The precipitate was filtered, washed with ethanol and crystallized from DMF/ethanol (3:1).

#### 3.1.17. 6-Amino-5-((4-(dimethylamino)benzylidene)amino)-1-methyl-2-thioxo-2,3-dihydropyrimidin-4(1H)-one [44] (**14**)

Yield: 96%; m.p. = 282–284 °C; IR (KBr) ν_max_ (cm^−1^): 3332, 3147 (NH_2_), 3101 (CH arom.), 2939 (CH aliph.), 1643, 1589 (C=O), 1527 (C=N), 1489 (C=C), 810 (*p*-substituted phenyl); ^1^H NMR (DMSO-d6) δ 12.11 (s,1H), 9.60 (s, 1H), 7.75–7.73 (d, *J* = 8.8 Hz, 2H, arom.), 7.28 (s, 2H), 6.74-6.72 (d, *J* = 8.9 Hz, 2H, arom.), 3.82 (s, 3H, CH_3_), 2.98 (s, 6H, 2CH_3_); ^13^C NMR (DMSO-d_6_): δ 172.87 (C=S), 155.34, 153.49 (C=O), 153.22, 151.45, 129.07, 126.03, 111.58, 103.59, 39.82, 36.32 ppm.; MS: m/z (%) = M+, 303 (36), 283 (89), 240 (43), 232 (50), 219 (100), 217 (45), 214 (47), 183 (53), 177 (66), 104 (45), 103 (44), 90 (85), 48 (51); Anal. calcd. for C_14_H_17_N_5_OS (303.38): C, 55.43; H, 5.65; N, 23.08; Found: C, 55.47; H, 5.84; N, 22.79.

#### 3.1.18. 6-Amino-1-benzyl-5-((4-(dimethylamino)benzylidene)amino)pyrimidine-2,4(1H,3H)-dione (**15**)

Yield: 86%; m.p. = 270–272 °C; IR (KBr) ν_max_ (cm^−1^): 3356, 3163 (NH_2_), 3032 (CH arom.), 2893 (CH aliph.), 1697, 1604 (C=O), 1550 (C=N), 1496 (C=C), 817 (*p*-substituted phenyl); ^1^H NMR (DMSO-d6) δ 10.79 (s, 1H), 9.55 (s,1H), 7.68-7.66 (d, *J* = 8.9 Hz, 2H, arom.), 7.37–7.34 (t, *J* = 7.3 Hz, 2H, arom.), 7.29–7.23 (m, 3H, arom.), 7.13 (s, 2H, NH_2_), 6.71–6.69 (d, *J* = 8.9 Hz, 2H, arom.), 5.19 (s,2H), 2.95 (s,6H).); ^13^C NMR (DMSO-d_6_): δ 158.15, 153.39, 151.16, 143.26, 136.35, 128.59, 128.44, 127.21, 126.65, 126.36, 111.68, 103.68, 99.37, 44.48, 35.98 ppm.; MS: m/z (%) = M+, 363 (35), 362 (33), 342 (48), 314 (100), 313 (47), 306 (40), 302 (58), 299 (78), 297 (58), 255 (71), 177 (44), 129 (65), 104 (62), 102 (54), 60 (37), 49 (55); Anal. calcd. for C_20_H_21_N_5_O_2_ (363.42): C, 66.10; H, 5.82; N, 19.27; Found: C, 66.24; H, 5.57; N, 19.13.

#### 3.1.19. 6-Amino-5-((4-(dimethylamino)benzylidene)amino)-1-ethylpyrimidine-2,4(1H,3H)-dione (**16**)

Yield: 78%; m.p = 280 °C; IR (KBr) ν_max_ (cm^−1^): 3379, 3170 (NH_2_), 3024 (CH arom.), 2939 (CH aliph.), 1689, 1604 (C=O), 1543 (C=N), 1496 (C=C), 810 (*p*-substituted phenyl); ^1^H NMR (DMSO-d_6_): δ 10.61 (s,1H), 9.54 (s, 1H), 7.69–7.67 (d, *J* = 8.8 Hz, 2H, arom.), 7.17 (s, 2H), 6.72–6.70 (d, *J* = 8.8 Hz, 2H, arom.), 3.94–3.92 (q, *J* = 6.9 Hz, 2H), 2.98 (s, 6H, 2CH_3_), 1.16-1.13 (t, *J* = 6.9 Hz, 3H); ^13^C NMR (DMSO-d_6_): δ 157.99, 152.98, 151.09, 150.70, 148.90, 128.49, 126.66, 111.70, 99.15, 36.97, 13.37, 13.10 ppm.; MS: m/z (%) = M+, 301 (44), 292 (34), 283 (40), 248 (39), 204 (34), 109 (37), 68 (100); Anal. calcd. for C_15_H_19_N_5_O_2_ (301.35): C, 59.79; H, 6.36; N, 23.24; Found: C, 59.93; H, 6.14; N, 23.37.

### 3.2. Biological Activity

#### 3.2.1. Antibacterial and Antifungal Activity

The biological activity of the synthesized uracil derivative compounds was assessed against various antibiotic resistant bacteria such as *E. coli*, *Pseudomonas* sp., *Salmonella* sp., *S. aureus* and *Bacillus* sp., isolated from different human medical specimens (Al-Ahrar Hospital, Zagazig, Egypt). These bacterial isolates were identified based on their biochemical features according to Bergey’s manual [61,62,63]. In addition, the activity of these compounds was evaluated against various pathogenic fungi; *A. flavus* and *C. albicans*. The antimicrobial activity of the tested compounds was determined by the disc-diffusion assay [64]. One mL of bacteria cell suspensions (10^7^ CFU/mL) from 24 h old cultures, was seeded into the nutrient agar medium, shacked vigorously then poured into sterilized Petri-dish. After medium solidification, 20 μL of the different concentrations of the synthesized compounds were loaded into filter paper disc (8.0 mm) that placed on the plate surface, then, the cultures were incubated for 37 °C for 24 h. The experimental compounds were completely dissolved in 10% DMSO, as well as, the further dilutions were made using 10% DMSO. The solubility and stability of the synthesized compounds were assessed from the UV-Vis spectral analysis. The compounds were completely dissolved and with no signs of precipitation over 5 days at room temperature (Figure S1). After incubation, observed for the zone of inhibition and diameter of these zones were measured in millimeters. The inhibition area was calculated comparing to the total bacterial growth area using Image J software portal [65].

#### 3.2.2. Transmission Electron Microscopic Analysis

For evaluation of the cytomorphological alterations induced by the most active compounds, the most resistant bacteria were treated with the compounds at their MIC doses, and the cellular organelles were investigated. The IC_50_ value was expressed by the concentration of compounds inhibiting the growth of 50% of initial microbial growth, as revealed from the regression curve. The Minimum Inhibitory Concentration (MIC) was expressed by the concentration of compound preventing the visible growth of microorganism. The IC_50_ value and MIC value of the potent compounds **3**, **12**, **13**, **14** and **16** were summarized in Table S6. The bacterial broth culture was incubated for 6 h, then amended with the tested compounds at their MIC dose, and continue for 24 h incubation at the same conditions. The bacterial cells were collected by centrifugation at 6000 rpm for 10 min, washed with sterile distilled water. The samples were prepared by immersing in primary fixative (2.5% glutaraldehyde buffered to pH 7.4 with 0.2 M phosphate buffer) for 3 h, and then post-fixed in 1 % osmium tetraoxide for 2 h, buffered with the phosphate buffer for 30 min. All steps of fixation were carried out at 4 °C, the samples were dehydrated in a series of ethanol (50% to 100%), then embedded in resin capsule, that sectioned into ultrathin section of 70 nm, loaded on copper grids and contrasted with uranium acetate and lead citrate prior examination by JEOL-1200 EX microscope (National Research Centre, Giza, Egypt).

#### 3.2.3. Cytotoxicity Assay

The cytotoxicity of the experimental compounds was evaluated towards VERO-E6 cells using the 3-(4,5-dimethylthiazol-2-yl)-2, 5-diphenyltetrazolium bromide (MTT) assay with slight modifications [64]. The stock solutions of compounds were prepared by dissolving in 10% DMSO in ddH_2_O and diluted further to the working solutions with DMEM. Briefly, the cells were seeded in 96 well-plates (100 µL/well at a density of 3 × 10^5^ cells/mL), incubated for 24 h at 37 °C in 5%CO_2_, the cells were treated with various concentrations of the tested compounds in triplicates, and further incubated for 24 h. The supernatant was discarded and cell monolayers were washed with sterile phosphate buffered saline and the MTT solution (20 µL of 5 mg/mL stock solution) was added to each well and incubated at 37 °C for 4 h followed by medium aspiration. The developed formazan crystals were dissolved in 200 µL of acidified isopropanol and measured at λ_540_ nm using a multi-well plate reader. To assess the half maximal cytotoxic concentration (CC_50_), the percentage of cytotoxicity compared to the untreated cells was determined with the following equation.

% cytotoxicity = ((absorbance of cells without treatment − absorbance of cells with treatment) × 100)/(absorbance of cells without treatment)

The plot of % cytotoxicity versus sampleconcentration was used to calculate the concentration which exhibited 50% cytotoxicity (CC_50_).

### 3.3. Molecular Docking Study

The structures of all tested compounds were modeled using the Chemsketch software (http://www.acdlabs.com/resources/freeware/ (accessed on 5 May 2021)). The structures were optimized and energy minimized using VEGAZZ software [66]. The optimized compounds were used to perform molecular docking to elucidate the anticancer activity against one fungal target protein which is acyl carrier protein domain from a fungal type I polyketide synthase (ACP), and two bacterial proteins including DNA gyrase (DNAg), and Baumannii penicillin-binding protein (PBP) to speculate the proposed antifungal and antibacterial activity for the most promising compounds. The three-dimensional structure of the molecular target was obtained from Protein Data Bank (PDB) (www.rcsb.org (accessed on 5 May 2021): (PBD: 2KR5, https://www.rcsb.org/structure/2KR5 (accessed on 5 May 2021), (PDB: 1KZN, https://www.rcsb.org/structure/1KZN (accessed on 5 May 2021), and (PDB: 3UDI, https://www.rcsb.org/structure/3UDI (accessed on 5 May 2021) respectively. The steps for receptor preparation included the removal of heteroatoms (water and ions), the addition of polar hydrogen, and the assignment of charge. The active sites were defined using grid boxes of appropriate sizes around the bound cocrystal ligands. The docking study was performed using Autodock vina [67] and Chimera for visualization [68]. All docking procedures and scoring were recorded according to our previous publications [69,70,71].

Docking procedures and scoring were recorded according to established protocols [68,72,73,74].

### 3.4. Pharmacokinetics and ADME Activity

To identify the biological targets for the most promising tested compound, we employed searching in database function integrated on Swiss institute Bioinformatics tools. Further on absorption, distribution, metabolism, and excretion “ADME” were calculated hypothetically.

### 3.5. Computational Method

The geometric parameters and energies were computed by density functional theory at the B3LYP/CEP-31G level of theory, using the GAUSSIAN 98W package of the programs [75], on geometries that were optimized at CEP-31G basis set. The high basis set was chosen to detect the energies at a highly accurate level. The atomic charges were computed using the natural atomic orbital populations. The B3LYP is the key word for the hybrid functional [76], which is a linear combination of the gradient functionals proposed by Becke [77] and Lee, Yang and Parr [78], together with the Hartree-Fock local exchange function [79]. UV spectra were recorded in Rigol, Ultra- 3000 series in Enzymology and Fungal Biotechnology Lab, Faculty of Science, Zagazig University.

## 4. Conclusions

The corresponding xanthines **3-5**, and imidazolone derivatives **6-13**, were obtained via reaction of oxazolone derivative **2** with 5,6-diaminouracils **1** under various conditions in excellent yields. The condensation of 5,6-diaminouracils **1** with aldehyde afforded 5-Benzylideneaminouracils **14-16.** The structural identities of the resulting compounds were resolved by IR, ^1^H-, ^13^C-NMR and Mass spectral analyses. The compounds **3, 6** and **16** displayed the highest activity against *E. coli* ((IC_50_ value 1.8–1.9 µg/mL). The compound **16** displayed a significant antifungal activity against *C. albicans* (0.82 µg/mL), *A. flavus* (1.2 µg/mL) comparing to authentic antibiotics. This study paves the way for new generations of uracil and imidiazolone derivatives as potential antimicrobial and biologically active agents.

## Figures and Tables

**Table 1:** Antimicrobial activity of the synthesized derivatives of **3-16** towards *C. albicans*, *A. flavus*, *E. coli*, *S. aureus*, *Pseudomonas* sp., and *Salmonella* sp.

	Diameter of Inhibition Zone (mm)						
	Fungi		Gram Negative Bacteria			Gram Positive Bacteria	
Cpd	*C. albicans*	*A. flavus*	*E. coli*	*Salmonella* sp.	*Pseudomonas* sp.	*S. aureus*	*Bacillus* sp.
10% DMSO	-	*-*	*-*	*-*	*-*	*-*	*-*
**3**	10 ± 0.8	2	4 ± 0.2	0.2 ± 0.01	0	2 ± 0.01	0.7 ± 0.01
**4**	2	0	2	0.1	0	1 ± 0.01	0.1
**5**	2	0	2	0	0	0.8 ± 0.01	0.8 ± 0.04
**6**	10 ± 0.6	2	4 ± 0.1	0	0	1 ± 0.01	0.9 ± 0.01
**7**	2	0	2	0	0	0	0
**8**	2	3	2	0	1.8 ± 0.01	0.2	0
**9**	5 ± 0.2	2	3± 0.3	0	0.9 ± 0.05	0	0.8 ± 0.03
**10**	4 ± 0.2	0	4 ± 0.23	0	0	0	0
**11**	6 ± 0.3	3	3 ± 0.3	0	0	0	0
**12**	8 ± 0.4	4	5 ± 0.22	0	1.5 ± 0.01	0.3	0
**13**	6 ± 0.2	3	4 ± 0.23	0	0	0.4	0.9 ± 0.01
**14**	2	2	2	0	1.9 ± 0.03	0	0
**15**	7 ± 0.5	3	3 ± 0.1	0.1 ± 0.01	1.8 ± 0.03	0	0.8 ± 0.03
**16**	12 ±0.3	10	7 ± 0.2	0.2 ± 0.01	1.9 ± 0.04	0.7	1.2 ± 0.04
Fluconazole	2.1 ± 0.1	2.9 ± 0.1	0	0	0	0	0
Bacimethrin			0.9 ± 0.2	0.6 ± 0.2	0.8 ± 0.2	0.9 ± 0.2	0.7 ± 0.2
Primsol			0.6 ± 0.09	0.5 ± 0.02	0.4 ± 0.02	0.3 ± 0.01	0.4 ± 0.3

**Table 2:** IC_50_ values of the tested compounds against the various multidrug resistance microorganisms.

	IC_50_ Values of the Synthesized Compounds						
	Fungi		Gram Negative Bacteria			Gram Positive Bacteria	
Cpd	*C. albicans*	*A. flavus*	*E. coli*	*Salmonella* sp.	*Pseudomonas* sp.	*S. aureus*	*Bacillu*s sp.
**3**	1.07	4.9	1.8	3.9	5.2	2.8	2.4
**4**	5.0	6.0	4.8	2.1	5.7	2.1	3.2
**5**	6.0	7.5	4.5	5.0	6.1	2.9	2.8
**6**	1.02	4.2	1.9	4.9	5.1	2.9	2.2
**7**	5.3	8.0	5.1	6.2	7.1	5.2	5.2
**8**	5.1	7.5	4.9	7.9	1.9	5.4	5.5
**9**	4.9	3.8	4.1	8.0	4.2	6.0	3.8
**10**	4.2	8.0	2.1	7.9	2.9	6.2	4.9
**11**	3.2	3.2	2.2	7.0	3.9	6.3	5.5
**12**	1.46	2.9	2.11	7.2	2.2	3.1	6.9
**13**	3.3	3.9	1.9	2.3	6.9	3.2	2.8
**14**	4.2	3.9	5.1	8.1	2.8	4.0	4.9
**15**	4.1	4.2	4.9	7.2	2.9	5.1	5.3
**16**	0.82	1.2	1.98	1.5	2.1	2.67	2.1
Fluconazole	0.6 ± 0.1	6.0 ± 0.1	0	0	0	0	0
Bacimethrin	0	0	3.9 ± 0.6	4.8 ± 0.1	5.1 ± 0.1	4.2 ± 0.1	3.9 ± 0.1
Primsol	0	0	4.3 ± 0.4	4.8 ± 0.1	4.6 ± 0.1	5.0 ± 0.1	4.3 ± 0.1

**Table 3:** Results of the docking study of compounds against fungal type I polyketide synthase (ACP), DNA gyrase, and Baumannii penicillin-binding protein (PBP) in comparison to the corresponding co-crystallized ligands.

Compounds	APC Protein (PDB: 2KR5)			
	Free Binding of Energy (Kcal/mol)	H-Bond		
		No of H-Bond	Amino Acid Residues	Length Å
**3**	−5.1	1	GLU65	2.261
**12**	−5.1	1	GLU65	2.348
**13**	−5.2	1	GLU65	2.358
**16**	−4.5	1	GLU65	1.984
**PNS**	−4.5	2	GLU65	2.265
			ASP71	2.559
**Compounds**	**DNAg Protein (PDB: 1KZN)**			
	**Free Binding of Energy (Kcal/mol)**	**H-Bond**		
		**No of H-Bond**	**Amino Acid Residues**	**Length Å**
**3**	−8.2	1	THR165	3.074
**12**	−7.2	2	ASP49	2.576
			ASN46	3.395
**13**	−7.4	2	ASP49	2.573
			ASN46	3.368
**16**	−7.1	**2**	ASP73	2.276
			ASN46	2.392
**CBN**	−9.3	4	ASN46	3.142
			ARG136	2.930
			ARG136	2.923
			ARG136	3.244
**Compounds**	**PBP Protein (PDB: 3UDI)**			
	**Free Binding of Energy (Kcal/mol)**	**H-Bond**		
		**No of H-Bond**	**Amino Acid Residues**	**Length Å**
**3**	−8.7	1	ASN674	2.006
**12**	−8.2	2	SER470	2.118
			THR672	2.797
**13**	−7.6	2	SER470	2.458
			THR672	3.040
**16**	−7.3	3	SER470	2.697
			SER487	3.278
			THR672	2.916
**PNS**	−7.2	5	TYR670	3.006
			TYR670	3.035
			SER434	3.078
			THR672	3.128
			THR672	2.310

**Table 4:** Physiochemical properties and drug-likeness.

Properties	Compound 12
Formula	C_17_H_17_N_5_O_3_
Molecular weight	339.35
Num. of heavy atoms	25
Num. of aromatic heavy atoms	12
Num. of rotatable bonds	3
Num. H-bond donors (HBD)	2
Num. H-bond acceptor (HBA)	4
Molar reactivity	103.26
Topological Polar Surface area (TPSA)	113.55
Lipophilicity (Log P)	2.04
Water solubility (Log S)	−2.39

**Table 5:** Equilibrium geometric parameters bond lengths (Å), bond angles (˚), dihedral angles (˚), total energy (k cal/mol), heat of formation (k cal/mol), Mulliken charges over atoms and dipole moment of the N-(1-(3-benzyl-2,6-dioxo-2,3,6,7-tetrahydro-1*H*-purin-8-yl)-2-(4-chlorophenyl) vinyl)acetamide, compound **3** by using DFT calculations.

**Bond Length (Å)**					
C1-C2 C2-C3 C3-C4 C4-C6 C5-C6 C1-C5 C1-Cl7 C4-C8 C8-C9 C9-C10 C9-N12	1.346 1.345 1.348 1.354 1.343 1.341 1.726 1.351 1.353 1.346 1.354	C13-N12 C13-O14 C13-C15 C10-N11 N11-C16 C16-C18 N17-C18 C10-N17 C16-C19 C19-N20 C19-O23	1.384 1.209 1.521 1.339 1.334 1.338 1.348 1.269 1.349 1.377 1.210	N20-C21 C21-N22 C21-O24 C18-N22 N22-C25 C25-C26 C26-C27 C27-C28 C28-C29 C29-C31 C30-C31 C26-C30	1.389 1.395 1.208 1.351 1.453 1.556 1.346 1.342 1.341 1.342 1.342 1.346
**Bond Angle (°)**					
C3C4C8 C6C4C8 C4C8C9 C8C9N12 C8C9C10	124.63 118.83 131.89 113.55 126.94	C9N12C13 N12C13C15 N12C13O14 O14C13C15 C9C10N17	124.18 122.51 118.39 119.04 125.31	C9C10N11 C18N22C25 C25N22C21 C26C25N22 C25C26C27 C25C26C30	123.47 120.23 120.71 113.98 119.35 122.79
**Dihedral Angles (°)**					
C3C4C8C9 C6C4C8C9 C4C8C9N12 C4C8C9C10 C8C9N12C13 O23C19N20C21	18.74 −165.54 −179.95 20.23 −105.95 178.85	C10C9N12C13 C9N12C13C15 N12C9C10N11 N12C9C10N17 C10N17C18N22 O24C21N20C19	82.58 −19.77 23.75 −154.23 178.05 179.53	C26C25N22C18 C26C25N22C21 N22C25C26C20 N22C25C26C27 C8C9N10N17	−84.71 87.88 −4.19 175.85 102.31
**Mulliken charges**					
C1 Cl7 N12 C13 C9 O14	0.237 −0.295 −0.284 0.424 0.114 −0.488	C10 N11 N17 C16 C18 N22	0.217 −0.107 −0.334 −0.111 0.246 −0.198	C21 N20 O24 C19 O23	0.501 −0.324 −0.432 0.425 −0.446
Total energy/k cal/mol Heat of formation k cal/mol Total dipole moment/D			−172,059.722 −10,607.049 3.078		

**Table 6:** Calculated charges on donating sites and energy values (HOMO, LUMO, Energy gap ∆E/eV, hardness (η), global softness (S), electro negativity (χ), absolute softness (σ), chemical potential (Pi), global electrophilicity (ω) and additional electronic charge (∆N_max_) of the studied four compounds by using DFT calculations.

Parameters	Compound 3	Compound 12	Compound 13	Compound 16
HOMO, H	−0.319	−0.321	−0.315	−0.309
LUMO, L	−0.237	−0.245	−0.254	−0.237
I = −H	0.319	0.321	0.315	0.309
A = −L	0.237	0.245	0.254	0.237
∆E = L − H	0.082	0.067	0.061	0.072
η = (I − A)/2	0.041	0.034	0.031	0.036
χ = −(H − L/2)	0.278	0.283	0.285	0.237
σ = 1/η	24.390	29.412	32.258	27.778
S = 1/2 η	12.195	14.706	16.129	13.889
Pi = −χ	−0.278	−0.283	−0.285	−0.237
ω = (Pi)^2^/2 η	0.942	1.178	1.310	0.780
∆N_max_ = χ/η	6.780	8.324	9.194	6.583

(I) is ionization energy. (A) is an electron affinity.

**Table 7:** Composition of the frontier molecular orbital for studied the compounds **3**, **12**, **13** and **16** by using DFT/B3LYP/Cep-31G.

	Compound 3			Compound 12	
	H	L		H	L
2,6-dioxopurine ring C10, N11, C16-O24	55.5%	53.7%	2,4-dioxo-6-aminopyrimidine ring C1-O8, N12	25.6%	0.0%
Chlorophenyl ring C1-Cl7	15.4%	21.8%	2-methyl-5-oxoimidazol ring N13-C19	61.2%	51.9%
Vinyl group C8-C9	7.9%	15.9%	Benzylidene ring C20-C26	13.2%	48.1%
acetamide group N12-C15	20.2%	8.6%	Ethyl group C9, C11	0.0%	0.0%
Benzyl group C25-C31	1%	0.0%			
	**Compound 13**			**Compound 16**	
	**H**	**L**		**H**	**L**
2-thio-4-oxo-6-aminopyrimidine ring C1-S8, N11	56.3%	0.0%	2,4-dioxo-6-aminopyrimidine ring C13-O19, N23	41.3%	19.5%
2-methyl-5-oxoimidazol ring N12-C18	41.9%	52.7%	dimethylaminobenzylidene ring C1-N9, C33-C34	33.6%	58.7%
Benzylidene ring C19-C25	1.8%	31.4%	Amino group N24-C25	25.1%	21.8%
Methyl group C9	0.0%	15.9%	Ethyl group C20, C22	0.0%	0.0%

**Table 8:** Computed excitation energies (eV), electronic transition configurations and wave lengths (nm) of the obtained stable compounds; **3** and **12** by using B3LYP/Cep-31G for the, (f ≥ 0.001) f = oscillator strengths compared with experimental data of UV-Visible spectra, λ_max_, nm.

Compound	eV	nm	Major Contributions	Assignment	λ_max_, nm
**3**	4.9546	250.24	H-11 → L(4.5%), H-11 → L + 6(2.9%), H → L(4.9%), H-7 → L(3.2%), H-5→L(34.5%), H-3 → L(3.7%), H-1 → L(14.7%), H → L(4.4%)	π-π *	265
	4.7874	258.98	H-11 → L (7.8%), H-10 → L (2.5%), H-5 → L (2.8%), H-1→L (78.4%), H → L(4,6%), H → L + 1(3.6%)	π-π *	275
	4.4144	280.86	H-11 → L + 7 (4.9%), H-10 → L + 6 (14.1%), H-10 → L + 7 (41.8%), H-7 → L + 7 (6.7%), H-6 → L + 6 (9.6%), H-6 → L + 7 (26.7%)	n-π *	290
	4.0996	302.43	H-9 → L (5.2%), H-9 → L + 5 (61.6%), H-9 → L + 6 (21.3%), H-9 → L + 7 (2.7%), H-8 → L + 5(6.3%), H-8 → L + 6 (2.8%)	π-π *	305
	3.4797	356.3	H-11 → L + 2 (5.3%), H-10 → L (6.3%), H-10 → L + 1 (10.4%), H-6 → L (22.9%), H-6 → L + 1 (38.3%), H-6 → L + 7 (8.8%), H-6 → L + 10 (7.8%)	n-π *	368
	3.2847	377.47	H-1 → L (4.5%), H → L(93.4%)	π-π *	383
**12**	4.7935	258.65	H-8 → L + 1 (2.4%), H-1 → L + 1 (54.4%), H → L + 1 (43%)	π-π *	269
	4.4183	280.61	H-6 → L (5.8%), H-4 → L (9.8%), H-3 → L (8.6%), H-2 → L(2.7%), H-1 → L(72.8%)	π-π *	278
	4.3485	285.12	H-9 → L + 3 (20.5%), H-9 → L + 4 (12.4%), H-8 → L + 3 (7.6%), H-8 → L + 4 (4%), H-7 → L + 3 (27.3%), H-3 → L + 4 (16.6%), H-5 → L + 3 (7%), H-5 → L + 4 (4.2%)	n-π *	300
	4.0172	308.64	H-10 → L (5%), H-8 → L (35.2%), H-8 → L + 3 (3.3%), H-8 → L + 4 (7.3%), H-3 → L (15.4%), H-3 → L + 3 (8.5%), H-3 → L + 4 (8.2%), H-3 → L + 5 (7%)	n-π *	315
	3.8482	322.23	H-6 → L + 1 (6.1%), H-5 → L + 1 (35.8%), H-5 → L + 3 (13.5%), H-5 → L + 6 (13.6%), H-4 → L + 1 (18.3%), H-4 → L + 3 (6.3%), H-4 → L + 6 (6%)	n-π *	322
	3.7319	332.23	H → L (100%)	π-π *	338
	3.6207	342.43	H-8 → L (14.5%), H-8 → L + 5 (8.9%), H-6 → L (3.3%), H-5 → L(11.7%), H-5 → L + 5 (3%), H-4 → L (5.7%), H-3 → L (36.7%), H-3 → L + 5 (3.5%)	n-π *	350

## Data Availability

All the data are included on this manuscript as well as enclosed supplementary data.

## Supplementary Materials

The following data are available online https://www.mdpi.com/article/10.3390/ijms222010979/s1.
