# Supplementary material for: Synthesis, In Silico Prediction and In Vitro Evaluation of Antimicrobial Activity, DFT Calculation and Theoretical Investigation of Novel Xanthines and Uracil Containing Imidazolone Derivatives
Source: Int J Mol Sci. 2021 Oct 12;22(20):10979. doi: 10.3390/ijms222010979 (PMC8539769; doi:10.3390/ijms222010979)
Supplement: Supplementary file 1 [file ijms-22-10979-s001.zip › ijms-1395579-supplementary.pdf]

Table S1: Equilibrium geometric parameters bond lengths (Å), bond angles (˚),dihedral angles (˚), total energy (k cal/mol) , heat of formation (k cal/mol), Mulliken charges over atoms and dipole moment of the 6-amino-5-(4-benzylidene-2-methyl-5-oxo-4,5-dihydro-1H-imidazol-1-yl)-1-ethylpyrimidine-2,4(1H,3H)-dione, compound **12** by using DFT calculations.

Bond length (Å)					
C1-C2	1.351	C1-N13	1.343	C17-C19	1.501
C2-N6	1.355	N6-C9	1.458	C15-C20	1.343
C2-N12	1.342	C9-C11	1.534	C20-C21	1.351
C5-N6	1.388	N13-C14	1.349	C21-C22	1.352
C5-N4	1.381	C14-O18	1.209	C22-C23	1.342
C5-O8	1.208	C14-C15	1.341	C23-C24	1.339
C3-N4	1.376	C15-N16	1.361	C24-C25	1.338
C3-O7	1.211	N16-C17	1.352	C25-C26	1.344
C1-C3	1.362	N13-C17	1.368	C21-C26	1.347
**Bond angle (˚)**					
C1C2N12	117.49	C29C1N13	121.55	C1N13C17	129.89
C1N13C14	130.72	N12C2N6	119.23	N12C2C1	117.49
N6C9C11	111.67	C21C20C15	136.14	C20C21C26	126.41
C20C21C22	117.68				
**Dihedral angles (˚)**					
C5N6C9C11	90.54	C11C9N6C2	-95.21	N12C2N6C9	21.27
C5N6C2N12	-164.45	N12C2C1C3	175.76	N12C2C1N13	-4.43
C17N13C1C2	-70.47	C17N13C1C3	109.33	N16C17N13C1	-166.79
C1N13C14C15	162.89	C1N13C14O18	-26.87	C26C21C20C15	7.63
C15C20C21C22	-176.18	N16C15C20C21	-171.89	C21C20C15C14	4.55
**Mulliken charges**					
C1	-0.136	N6	-0.208	N13	-0.135
C2	0.305	O7	-0.442	C14	0.392
C3	0.428	O8	-0.432	O18	-0.425
N4	-0.328	C9	0.072	N16	-0.385
C5	0.509	N12	-0.359	C15	0.068
C17	0.265				
Total energy/ k cal/mol		-141856.224			
Heat of formation k cal/mol		-8661.756			
Total dipole moment/D		8.326			

Table S2: Equilibrium geometric parameters bond lengths (Å), bond angles (˚),dihedral angles (˚), total energy (k cal/mol), heat of formation (k. cal/mol), Mulliken charges over atoms and dipole moment of the 6-amino-5-(4-benzylidene-2-methyl-5-oxo-4,5-dihydro-1H-imidazol-1-yl)-1-methyl-2-thioxo-2,3-dihydropyrimidin-4(1H)-one, compound **13** by using DFT calculations.

Bond length (Å)					
C1-C2	1.347	C9-N6	1.451	C14-C19	1.342
C2-N6	1.348	C5-S8	1.692	C19-C20	1.351
C5-N6	1.347	C1-N12	1.342	C20-C21	1.352
C5-N4	1.383	N12-C13	1.348	C21-C22	1.343
C3-N4	1.369	C13-C14	1.340	C22-C23	1.339
C1-C3	1.362	C14-N15	1.359	C23-C24	1.341
C3-O7	1.209	C16-N15	1.339	C24-C25	1.343
C2-N11	1.339	C16-C18	1.501	C20-C25	1.347
Bond angle (˚)					
C9N6C5	122.87	C9N6C2	121.44	C1C2N11	120.22
N11C2N6	118.79	N6C5S8	121.95	N4C5S8	119.41
O7C3N4	119.25	C7C3C1	120.69	N11C2C1	120.22
N12C1C2	122.62	N12C1C3	121.65	C1N12C13	129.48
C1N12C16	130.53	C18C16N15	122.15	C20C19C14	134.51
C18C16N12	124.29	C19C14C13	133.65	C19C20C21	118.23
Dihedral angles (˚)					
C9N6C5N4	119.63	C9N6C2C1	-125.36	C5N6C2N11	49.59
C9N6C5S8	-43.49	C3N4C5S8	-174.89	C2N6C5S8	153.03
N11C2N6C5	-146.64	N11C2C1C3	175.95	O7C3C1C2	169.59
N12C1C3O7	-13.93	C3C1N12C6	110.99	C3C1N12C13	-49.18
C13C14C19C20	6.42	C14C19C20C21	-173.59	C14C19C20C25	11.35
Mulliken charges					
C1	-0.118	N6	-0.163	N12	-0.134
C2	0.296	O7	-0.413	C13	0.393
C3	0.423	S8	-0.616	C14	0.064
N4	-0.261	C9	0.019	N15	-0.349
C5	0.478	N11	-0.359	C16	0.264
C18	-0.201				
Total energy/ k cal/mol			-132805.565		
Heat of formation k cal/mol			-8086.449		
Total dipole moment/D			8.586		

Table S3: Equilibrium geometric parameters bond lengths (Å), bond angles (˚),dihedral angles (˚), total energy (k cal/mol) , heat of formation (k cal/mol), Mulliken charges over atoms and dipole moment of the 6-amino-5-((4-(dimethylamino)benzylidene)amino)-1-ethylpyrimidine-2,4(1H,3H)-dione, compound **16** by using DFT calculations

.

Bond length (Å)					
C1-C2	1.347	N9-C34	1.457	C14-N15	1.376
C2-C3	1.343	C1-C25	1.346	C12-C14	1.361
C3-C4	1.342	C25-N24	1.347	C13-N23	1.343
C4-C5	1.342	C12-N24	1.351	N17-C20	1.459
C5-C6	1.343	C12-C13	1.352	C20-C22	1.534
C1-C6	1.347	C13-N17	1.367	C16-O19	1.208
C4-N9	1.351	N17-C16	1.388	C14-O18	1.211
N9-C33	1.457	C16-N15	1.379		
Bond angle (˚)					
C33N9C34	119.99	C34N9C4	120.05	C33N9C4	119.99
C3C4N9	120.14	N9C4C5	120.26	C6C1C25	119.92
C2C1C25	121.87	C1C25N24	121.79	C25N24C12	120.42
N24C21C13	118.49	N24C12C14	123.63	N23C13N17	119.95
N23C13C12	116.15	C20N17C13	121.78	C20N17C16	119.12
N17C20C22	110.71	C12C14O18	122.35	O18C14N15	117.69
Dihedral angles (˚)					
C13N17C20C22	-83.59	C33N9C4C3	-73.95	C25N24C12C14	50.26
C20N17C16O19	1.65	C34N9C4C5	-76.79	C25N24C12C13	-131.88
C20N17C13C12	178.47	C22C20N17C16	93.05	N24C25C1C6	179.69
N24C12C14O18	0.10	C20N17C13N23	-2.51	C33N9C4C5	106.00
C1C25N24C12	-179.58	N23C13C12N24	0.60	C34N9C4C3	103.49
N24C25C1C2	-0.510				
Mulliken charges					
C12	0.129	N17	-0.203	N9	-0.437
Cl3	0.298	O18	-0.448	C33	0.032
C14	0.424	O19	-0.459	C34	0.033
N15	-0.429	C20	0.069	C25	0.071
C16	0.506	C4	0.186		
Total energy/ k cal/mol			-123990.259		
Heat of formation k cal/mol			-8026.826		
Total dipole moment/D			6.051		

Table S4: Computed excitation energies (eV), electronic transition configurations and wave lengthes (nm) of the obtained stable compounds; **13** and **16** by using B3LYP/Cep-31G for the, (f≥ 0.001) f= oscillator strengths compared with experimental data of UV-Visible spectra, λ_{max}, nm.

Compound	eV	nm	Major Contributions	Assignment	λ_{max}, nm
**13**	4.7546	260.77	H-11→L+1(19.8%), H-11→L+2(3.7%), H-8→L+1 (9.7%), H-7→L+1(3.7%), H-7→L+2(4.9%), H-3→L+1(31.1%), H-3→L+2(3.8%), H→L+1 (16.1%)	π-π*	267
	4.7117	263.14	H-7→L (2.6%), H-6→L (5.2%), H-5→L (15.7%), H-4→L (3.5%), H-3→L (49%), H-3→L(2.5%), H-1→L (2.5%), H-1→L+4(2.2%)	π -π*	
	4.3637	284.13	H-8→L+1 (4.1%), H-3→L (2.7%), H-3→L+1 (3.2%), H-3→L+2 (32.4%), H-1→L+2 (16.2%), H→L+1 (8%), H→L+2(32.9%)	π-π*	290
	4.2944	288.71	H-10→L (8.2%), H-10→L+4 (2.5%), H-9→L (25%), H-3→L+1 (4.8%), H-9→L+4(7.8%), H-6→L(3.1%), H-6→L+4 (4.5%), H-3→L+4 (2.9%), H-5→L (17.2%), H-5→L+4(18.8%), H-5→L+5(4.5%)	n-π*	
	3.886	319.05	H-11→L+9 (2.4%), H-1→L+1 (86.7%), H-1→L+2 (7.6%), H→L+1 (3.1%)	π -π*	322
	3.7876	326.03	H-7→L+1 (40.5%), H-7→L+2(22.4%), H-7→L+5 (3%), H-7→L+6 (21.6%), H-6→L+1 (5.4%), H-6→L+2(3.4%)	n-π*	
	3.7785	328.13	H→L (100%)	π-π*	
	3.6527	339.43	H-10→L (2.8%), H-9→L(12.7%), H-9→L+4 (8.1%), H-6→L (9.7%), H-6→L+5(8.5%), H-5→L (4.1%), H-5→L+5 (10.7%), H-4→L (82.6%), H-2→L+2 (17.3%)	n-π*	339
	3.4525	359.12	H-2→L+1 (3.2%), H-3→L (7.8%)	n-π*	357
**16**	4.9616	249.89	H-1→L (8.6%), H→L+1 (6.1%), H-1→L+1 (3.2%), H→L+1 (47.7%), H-1→L+4 (4.9%), H-1→L (29.2%)	π-π*	260
	4.4466	278.83	H-8→L+3 (29.2%), H-7→L+3 (26.6%), H-6→L+3 (30.8%), H-5→L+3 (9.8%), H-3→L+3 (3.3%)	n-π*	291
	3.9555	313.44	H-8→L (9.7%), H-6→L (12.1%), H-3→L (4.8%), H-2→L (14.5%), H-1→L (21.4%), H→L (37.1%)	π-π*	321
	3.6964	335.42	H-8→L+1 (6.1%), H-5→L+1 (37.1%), H-5→L+3 (10.2%), H-5→L+5 (9%), H-3→L+1 (20.4%), H-3→L+3 (5%), H-3→L+5 (4.2%), H-2→L+1 (7.6%)	π-π*	345
	3.3903	365.71	H-8→L (7.2%), H-6→L (11.7%), H-3→L (3%), H-2→L (3.3%), H-1→L (4.2%), H→L (66.4%), H→L+4 (3.8%)	π-π*	376
